# Inference of chromosomal inversion dynamics from Pool-Seq data in natural and laboratory populations of *Drosophila melanogaster*

**DOI:** 10.1111/mec.12594

**Published:** 2013-12-20

**Authors:** Martin Kapun, Hester van Schalkwyk, Bryant McAllister, Thomas Flatt, Christian Schlötterer

**Affiliations:** * Institut für Populationsgenetik, Vetmeduni ViennaVeterinärplatz 1, Vienna, A-1210, Austria; † Vienna graduate school of Population Genetics; ‡ Department of Biology, University of IowaIowa City, IA, 52242, USA

**Keywords:** experimental evolution, genomics, inversions, Pool-Seq, population genetics

## Abstract

Sequencing of pools of individuals (Pool-Seq) represents a reliable and cost-effective approach for estimating genome-wide SNP and transposable element insertion frequencies. However, Pool-Seq does not provide direct information on haplotypes so that, for example, obtaining inversion frequencies has not been possible until now. Here, we have developed a new set of diagnostic marker SNPs for seven cosmopolitan inversions in *Drosophila melanogaster* that can be used to infer inversion frequencies from Pool-Seq data. We applied our novel marker set to Pool-Seq data from an experimental evolution study and from North American and Australian latitudinal clines. In the experimental evolution data, we find evidence that positive selection has driven the frequencies of *In*(*3R*)*C* and *In*(*3R*)*Mo* to increase over time. In the clinal data, we confirm the existence of frequency clines for *In(2L)t*, *In(3L)P* and *In(3R)Payne* in both North America and Australia and detect a previously unknown latitudinal cline for *In(3R)Mo* in North America. The inversion markers developed here provide a versatile and robust tool for characterizing inversion frequencies and their dynamics in Pool-Seq data from diverse *D. melanogaster* populations.

## Introduction

Inversions are common chromosomal variants of great evolutionary interest; they arise from structural mutations, which cause a reversal of gene order relative to the standard chromosomal arrangement. They have, for example, been found to be involved in sex chromosome evolution (McAllister 2003; Charlesworth *et al.* 2005) and may be key factors for speciation (Noor *et al.* 2001; Rieseberg 2001; Hey 2003; Manoukis *et al.* 2008; Neafsey *et al.* 2010). Due to early efforts by Dobzhansky and his co-workers, much of our current understanding of the genetics and evolution of inversion polymorphisms comes from work on species of the genus *Drosophila* (Dobzhansky 1971; Powell 1997). Inversion polymorphisms are pervasive within numerous *Drosophila* species, and a large body of classical work suggests that they are key drivers of evolutionary dynamics and adaptive change in natural populations (for reviews, see Krimbas & Powell 1992; Hoffmann *et al.* 2004; Faria & Navarro 2010).

Several lines of evidence indicate that selection plays a key role in maintaining inversion polymorphisms and in shaping their frequencies in natural populations. First, the frequencies of specific inversion polymorphisms in *Drosophila* have been correlated with numerous life history, physiological and morphological traits (for reviews, see Hoffmann *et al.* 2004; Hoffmann & Rieseberg 2008). Second, numerous polymorphic inversions show strongly clinal (e.g. latitudinal) patterns of variation, and many of these patterns are replicated across continents in broadly distributed *Drosophila* species, including *D. subobscura* (Prevosti *et al.* 1985, 1988; Krimbas & Powell 1992), *D. melanogaster* (Mettler *et al.* 1977; Knibb *et al.* 1981; Knibb 1982), *D. pseudoobscura* (Dobzhansky & Epling 1944; Dobzhansky 1971; Anderson *et al.* 1991; Powell 1997) and *D. robusta* (Etges & Levitan 2004). In addition, similarly, persistent longitudinal clines have been identified for *Anopheles* species in Africa (Cheng *et al.* 2012). Third, analyses of latitudinal gradients repeated over time indicate that many of these clines remain stable (Anderson *et al.* 1987) or that they shift with latitude over many years (Anderson *et al.* 2005). Finally, the fitness advantage and the dynamics of inversion heterokaryotypes have been monitored both in natural populations and under laboratory conditions, and the results are often consistent with selection shaping inversion dynamics (Wright & Dobzhansky 1946; Dobzhansky 1971). Moreover, inversions effectively suppress recombination around inverted regions in heterokaryotypes (Sturtevant 1917). Although double cross-over and gene conversion can maintain a limited amount of gene flux between inverted and noninverted arrangements (Chovnick 1973; Rozas & Aguadé 1994; Betrán *et al*. 1997; Schaeffer & Anderson 2005), inversions typically cause a pattern of cryptic, chromosome-specific population substructure (Navarro *et al.* 2000). However, despite the large body of work on the population genetics of inversion polymorphisms (Charlesworth & Charlesworth 1973; Charlesworth 1974), the nature of variation harbored by inversions and the molecular targets of selection within inversions remain very poorly understood to date (Kirkpatrick & Barton 2006; Hoffmann & Rieseberg 2008).

Several recent studies have used next-generation sequencing (NGS) technology to obtain individual-based whole-genome sequence information from multiple individuals and to use such information to analyse the details of inversion breakpoint structure, the evolutionary age of inversions and the patterns of genetic variation associated with inversions in natural populations with previously unprecedented resolution (Corbett-Detig & Hartl 2012; Corbett-Detig *et al.* 2012; Langley *et al.* 2012). However, due to the still relatively high costs associated with sequencing many individuals, the availability of whole-genome population data for multiple individuals remains limited today. A widely used, very simple and cost-effective alternative is to sequence pools of DNA from multiple individuals (‘Pool-Seq’; Futschik & Schlötterer 2010), but an obvious drawback of this approach is that it does not yield haplotype information and thus precludes the direct estimation of inversion frequencies.

Given the widespread use of the Pool-Seq method in molecular population genomics (Burke *et al.* 2010; Turner *et al.* 2010, 2011; Kolaczkowski *et al.* 2011; Fabian *et al.* 2012; Orozco-ter Wengel *et al.* 2012; Tobler *et al.* 2013), and given the importance of inversions in shaping patterns of molecular variation in natural populations, here we have developed a novel set of SNP markers for seven cosmopolitan inversions in *D. melanogaster* (i.e. *In(2L)t*, *In(2R)Ns, In(3L)P, In(3R)C, In(3R)K, In(3R)Mo, In(3R)P*). By applying this new marker set to several natural and experimental populations, we demonstrate that inversion frequencies and their dynamics can be reliably estimated from and examined with Pool-Seq data.

## Materials and methods

We first developed a set of inversion-specific marker SNPs by karyotyping and whole-genome sequencing of individuals from an ongoing experimental evolution study in our laboratory (see Orozco-ter Wengel *et al.* 2012; Tobler *et al.* 2013 results). To supplement this analysis, we also used haplotype information from the *Drosophila* Population Genomics Project (DPGP, DPGP2) (Langley *et al.* 2012; Pool *et al.* 2012; http://www.dpgp.org; for details, see below).

### Experimental evolution populations

In brief, we carried out an experimental evolution experiment (‘laboratory natural selection’, LNS) using an outbred base population of *D. melanogaster* derived from 113 isofemale lines isolated from a wild population from Povoa de Varzim (Northern Portugal) in 2008 (see Orozco-ter Wengel *et al.* 2012; Tobler *et al.* 2013 for details). We exposed three replicate populations per treatment to two thermal selection regimes, with temperatures changing every 12 h between 18 and 28 °C (‘hot’) and between 10 and 20 °C (‘cold’). In both treatments, replicate populations were maintained with discrete generations at a fixed population size of 1000 individuals per replicate.

### Karyotyping

To determine the distribution of inversions in the above-mentioned selection experiment, we karyotyped sample individuals from the experimental populations. We randomly chose males of unknown chromosomal karyotype from three different cohorts: (i) isofemale lines, which were initially used to establish the base population of the experimental evolution experiment; (ii) three replicate populations from the ‘cold’ treatment at generation 34 of selection; and (iii) three replicate populations from the ‘hot’ treatment at generation 60 of selection. Males were crossed to virgin females of a mutant strain (*y*[1]; *cn*[1] *bw*[1] *sp*[1]) homozygous for standard arrangement chromosomes. In the F1, we prepared polytene chromosome squashes from salivary glands of third instar larvae reared at 18 °C using orcein staining following standard protocols (Kennison 2000). Chromosome preparations were analysed using a Leica DM5500B microscope (Leica, Wetzlar, Germany). We determined chromosomal arms using reference maps in Bridges (1935); inversion loops in heterokaryons were identified from reference photographs in Ashburner & Lemeunier (1976). Corpses of some larvae used for chromosome preparations were stored in 96% EtOH for later DNA extraction and sequencing (Table 1).

**Table 1:** Inversion counts and frequencies. Counts and frequencies (in parentheses) of six inversions identified by karyotyping in the base population and three replicate populations in each selection regime. The sample size *n* refers to the number of chromosomes sampled from each population

Population	*n*	*In(2L)t*	*In(2R)Ns*	*In(3L)P*	*In(3R)P*	*In(3R)Mo*	*In(3R)C*
Base	37	12 (0.32)	2 (0.05)	1 (0.03)	4 (0.11)	4 (0.11)	5 (0.14)
Cold - R1	36	13 (0.36)	0 (0)	3 (0.08)	3 (0.08)	7 (0.19)	2 (0.06)
Cold - R2	45	4 (0.09)	0 (0)	2 (0.04)	0 (0)	12 (0.27)	12 (0.27)
Cold - R3	30	10 (0.33)	2 (0.07)	0 (0)	0 (0)	6 (0.2)	3 (0.1)
Hot - R1	42	15 (0.36)	0 (0)	2 (0.05)	0 (0)	2 (0.05)	19 (0.45)
Hot - R2	44	10 (0.23)	0 (0)	3 (0.07)	2 (0.05)	1 (0.02)	15 (0.34)
Hot - R3	41	16 (0.39)	0 (0)	0 (0)	0 (0)	1 (0.02)	17 (0.41)
Sum	275	80	4	11	9	33	73

### Single individual sequencing

Based on information from our karyotyping, we selected 15 corpses of F1 larvae from three replicate populations of the hot and the cold selection regime at generations 60 and 34, respectively, for whole-genome sequencing (Table S1). We prepared individual genomic libraries by extracting DNA from homogenized single larval carcasses using the Qiagen DNeasy Blood and Tissue Kit (Qiagen, Hilden, Germany) and sheared DNA with a Covaris S2 device (Covaris Inc., Woburn, MA, USA). To identify residual heterozygosity in the reference strain (*y*[1]; *cn*[1] *bw*[1] *sp*[1]), we sequenced a pool of 10 adult females. Each library was tagged with unique 8-mer DNA labels and pooled prior to preparation of a paired-end genomic library using the Paired-End DNA Sample Preparation Kit (Illumina, San Diego, CA, USA); each library was sequenced on a HiSeq2000 sequencer (Illumina) (2 × 100 bp paired-end reads).

### Mapping of reads

Raw reads were trimmed to remove low-quality bases (minimum base quality: 18) using *PoPoolation* (Kofler *et al.* 2011) and mapped against the *D*. *melanogaster* reference genome (v.5.18) and *Wolbachia* (NC_002978.6) with *bwa* (v.0.5.7; Li & Durbin 2009) using the following parameters: –n 0.01 (error rate), -o 2 (gap opening), -d12, -e 12 (gap length) and -l 150 (disabling the seed option). We used the *bwa* module sample to reinstate pair-end information using Smith–Waterman local alignment. Using *samtools* (Li *et al.* 2009), we merged SAM files filtered for proper pairs with a minimum mapping quality of 30 in a mpileup file and used *Repeatmasker* 3.2.9 (www.repeatmasker.org) to mask simple repetitive sequence and transposable elements (based on the annotation of the *D. melanogaster* genome v. 5.34). Using *PoPoolation*, we masked all indels (and five nucleotides flanking them on either side) present in at least one population and supported by at least two reads to avoid confounding effects of mismapping reads containing indels. We excluded heterochromatic parts of chromosomes as well as reads mapping to the mitochondrial and *Wolbachia* genomes from further analyses.

### Reconstitution of chromosomal haplotypes

We used custom software tools to reconstruct paternal haplotypes from the sequenced F1 larvae (see above). By contrasting polymorphisms present in the F1 larvae to the reference sequence, we inferred paternal alleles at heterozygous sites in F1 hybrids. Polymorphic positions (minimum minor allele frequency >10%) in reads from the reference strain (see above) were excluded. In addition, we used the following criteria to avoid false-positive paternal alleles or false-negative maternal alleles during haplotype reconstruction: (i) we excluded positions with a minimum coverage <15 to reduce false negatives due to large sampling error; (ii) we calculated genome-wide coverage distributions for each F1 hybrid and each chromosomal arm separately and excluded positions with a coverage higher than the 95% percentile of the corresponding chromosomal arm to minimize false positives due to mapping errors and duplications; (iii) we only included alleles with a minimum count of 20 across all larvae sequenced; (iv) for SNPs with more than two alleles we only considered the two most frequent alleles; and (v) we only retained alleles for which the allele counts fell within the limits of a 90% binomial confidence interval based on an expected frequency of 50%. The efficiency of our SNP calling was evaluated using two different methods (see Supporting Information).

### Fixed differences associated with inversions

We took advantage of a worldwide sample of haplotypes originating from Africa, Europe and North America with known karyotype (Langley *et al.* 2012; Pool *et al.* 2012) and combined them with our haplotype data. In total, we compared 167 chromosomes from Africa (DPGP2; 107 individuals), Portugal (present study; 15 individuals), France (DPGP2; eight individuals) and USA (DPGP; 37 individuals [consensus genomes]) with known karyotypes, overall representing seven different inversions (*In(2L)t*, *In(2R)Ns, In(3L)P, In(3R)C, In(3R)K, In(3R)Mo, In(3R)P*) plus standard chromosome arrangements (Table S2). For each inversion type, we searched for fixed differences in the combined data set between inverted karyotypes and all other arrangements (i.e. standard arrangements and overlapping inversions) on the corresponding chromosome to identify inversion-specific SNP markers. We excluded positions where <80% of all individuals per arrangement were informative. We tested our method as described in the Supporting Information.

### Inversion frequency estimates

We used inversion-specific fixed differences between arrangements as SNP markers to estimate inversion frequencies from Pool-Seq data sets of Fabian *et al*. (2012; North American cline), Kolaczkowski *et al*. (2011; Australian cline), Orozco-ter Wengel *et al*. (2012; experimental evolution experiment, ‘hot’ selection regime) and Tobler *et al*. (2013; experimental evolution, ‘cold’ regime). Inversion frequencies were estimated from the average of all marker allele frequencies specific to a particular inversion. To reduce the variance in frequency estimates caused by sampling error, we excluded all positions with <10-fold coverage for all data sets except for the Australian data, where – given the generally low coverage in this data set – we chose a minimum coverage threshold of threefold. We also excluded all positions with coverage larger than the 95% percentile of the genome-wide coverage distribution to avoid errors due to mismapping or duplications. To evaluate the statistical significance of inversion frequency differentiation over time in our experimental evolution study, we integrated SNP-wise allele frequency information from three replicate populations in each selection regime across multiple time points by performing Cochran–Mantel–Haenszel tests (CMH; Landis *et al.* 1978) for each marker SNP separately and by averaging *P*-values across all tests. As replicates were not available for the two latitudinal data sets, we performed Fisher's exact tests (FET; Fisher 1922) on inversion frequency differences between the lowest latitude population and all other populations along each cline (North America, Australia) and combined *P*-values across all marker SNPs. We also compared inversion frequency estimates obtained from SNP markers to our empirical results from karyotyping as described in the Supporting Information. In addition, we also estimated inversion frequencies from our karyotype data and tested for significant differences in inversion frequency between the ‘hot’ and ‘cold’ selection regimes using the following fully factorial fixed-effects two-way anova model: *y *= *I *+ *T *+ *I *× *T*, where *y* denotes the inversion frequency, *I* the inversion type and *T* the selection regime using JMP (v.10.0.0; SAS Institute Inc., Cary, NC, USA).

### Genetic variation within inversions

To estimate genetic variation associated with each chromosomal arrangement, we estimated π in 100-kb nonoverlapping sliding windows for all chromosomes with the same karyotype. We excluded *In(2R)Ns* and *In(3R)P* from this analysis because both inversions were present in only one F1 larva of the 15 sequenced individuals. To compare π among arrangements, we randomly subsampled noninverted chromosomes to match the number of inverted chromosomes for *In(2L)t* and *In(3L)P*. For the inversions on *3R* (*In(3R)Mo* and *In(3R)C*), we were unable to subsample because our data set only contained three chromosomes with standard arrangement on this chromosomal arm. We therefore used all three individual chromosomes to estimate π and *F*_ST_ among chromosomal arrangements on *3R*. In addition, based on our estimates of π, we calculated *F*_ST_ between inverted and standard arrangement haplotypes in 100-kb nonoverlapping windows to measure the amount of chromosome-wide differentiation among arrangements.

### Linkage disequilibrium within inversions

For each chromosomal arm and arrangement, we estimated linkage disequilibrium (LD) by calculating *r*^*2*^ (Hill & Robertson 1968). We randomly sampled 5000 polymorphic SNPs along each chromosomal arm and visualized chromosome-wide pairwise *r*^*2*^ values using heat maps generated from the ‘LDHeatmap’ package (Shin *et al.* 2006) in *R* (R Development Core Team 2009). To quantify the difference in overall LD within noninverted and inverted chromosomes, we averaged all *r*^*2*^ values obtained from within the inverted regions for both standard and inverted haplotypes separately and calculated their ratios. As *r*^*2*^ depends strongly on the number of haplotypes, we always matched the number of inverted and standard chromosomes by subsampling the more frequent chromosomal arrangement.

### Expected inversion frequency change under neutrality

To estimate the degree to which inversion frequency changes observed during experimental evolution may be explained by drift alone, we employed forward simulations using a simple Wright–Fisher model of neutral evolution (Otto & Day 2007). For computations, we considered an inversion to represent allele *A*. Inversion frequencies p_0_(*A*) at the beginning of the experiment were obtained from frequency estimates based on our marker SNP approach. Additionally, we used estimates of the effective population size computed from real data of the LNS experiment and performed simulations using a value of 200 for the parameter *N* (Orozco-ter Wengel *et al.* 2012). Using 100 000 iterations, we simulated all three replicate populations for each temperature regime and using the same number of generations and inversion frequency as in the base population. We computed the empirical *P*-value by determining the number of simulations in which the polarized frequency change in each of the replicates was larger than in the observed data.

## Results

### Impact of inversions on genetic variation

In total, we identified six polymorphic cosmopolitan inversions segregating in our experimental evolution experiment: four common inversions (*In(2L)t, In(2R)Ns, In(3L)P*, *In(3R)Payne*) and two rare cosmopolitan inversions (*In(3R)Mo*, *In(3R)C*) (Mettler *et al.* 1977; Lemeunier & Aulard 1992) by cytological analysis of 275 polytene chromosomes from crosses of males with unknown karyotype and females of a noninverted mutant strain (see Table 1 and Table S3). We first aimed to examine the partitioning of genetic variation among inversions and standard chromosomes by performing whole-genome sequencing of 15 of 275 karyotyped individuals and by reconstructing the paternal haplotypes of these flies (see Materials and methods; Table S1; for the average sequencing depth of the individual DNA libraries, see Fig. S1). We estimated nucleotide diversity (π) and LD (*r*^*2*^) for both inverted and noninverted chromosomes and calculated pairwise *F*_ST_ to estimate genetic differentiation between arrangements. As *In(2R)Ns* and *In(3R)P* were only represented by one chromosome in our data, we did not analyse these inversions.

*2L*: π was similar between the standard arrangement and *In(2L)t* except for the breakpoint regions, where inverted chromosomes were less variable than the standard arrangement. *F*_ST_ was markedly higher within the inversion breakpoints as compared to outside of the inverted region (see Fig. S2a), but did not show distinct peaks at the putative breakpoints. Pairwise *r*^*2*^ values along *2L* indicated the existence of elevated LD in two regions located within the inversion and at the telomeric end of the chromosomal arm in haplotypes carrying *In(2L)t*. LD within inverted haplotypes was 2.46 times higher within the chromosomal region of the inversion as compared to standard arrangement chromosomes (see Fig. S3a).

*3L*: In contrast to standard arrangement chromosomes, we found reduced variability (π) around the proximal breakpoint of *In(3L)P* and in two large regions within the inversion as well as downstream of the distal breakpoints in chromosomes carrying the inverted arrangement. Although *F*_ST_ was homogenous along the chromosome, we detected an unusual haplotype structure in the *In(3L)P* chromosomes, with very large areas of pronounced LD within the inversion and also extending beyond it (see Figs S2b and S3b). Overall, LD within inverted haplotypes was approximately 4.7 times higher than in standard chromosomes.

**Figure 1:**
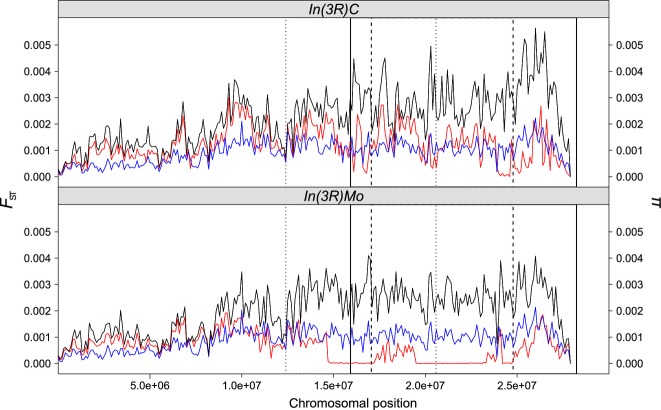
Nucleotide diversity (π) and genetic differentiation (*F*_ST_) for *In(3R)Mo* and *In(3R)C*. Line plots show nucleotide diversity (π) in standard (blue) and inverted (red) chromosomal arrangements; additionally, *F*_ST_ values (black) show the amount of genetic differentiation between arrangements. *In(3R)Mo* is based on five individuals and *In(3R)C* on six individuals. Values for standard arrangement chromosomes (blue) were obtained from comparing three individual chromosomes. Putative boundaries of the three overlapping inversions on *3R* are indicated by vertical black lines: the dashed line represents *In(3R)Mo*, the dotted line *In(3R)P* and the solid line *In(3R)C*.

**Figure 2:**
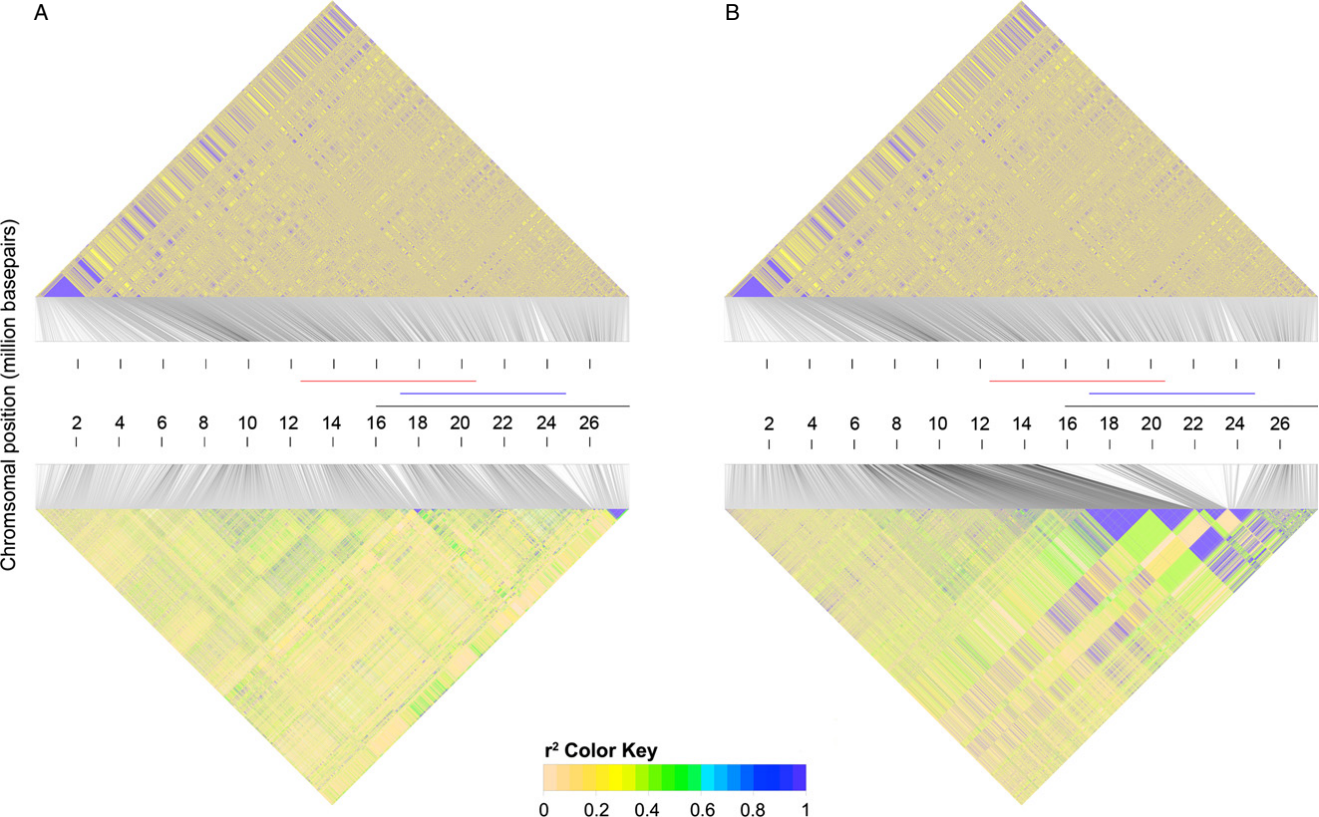
Linkage disequilibrium for *In(3R)Mo* and *In(3R)C*. Triangular heatmaps show estimates of *r*^*2*^ for 5000 randomly sampled SNPs across *3R*. The bottom triangles show the results for inverted arrangements, whereas the top triangles show the standard arrangements (based on three individuals). (A) *r*^*2*^ plots for *In(3R)Mo* (based on five individuals). (B) *r*^*2*^ plots for *In(3R)C* (based on six individuals). The chromosomal position of the three overlapping inversions on *3R* is indicated by a coloured line: *In(3R)P* (red), *In(3R)Mo* (blue) and *In(3R)C* (black).

*3R*: We found four chromosomal arrangements on the right arm of the third chromosome segregating in the populations from the selection experiment (standard arrangement, *In(3R)C*, *In(3R)Mo*, *In(3R)Payne*, all of which are known to overlap; Lemeunier & Aulard 1992). In contrast to chromosomes carrying *In(3R)C* and *In(3R)Mo*, the standard arrangement chromosomes did not exhibit any regions of reduced heterozygosity (Fig. 1). *In(3R)Mo* karyotypes harbored almost no genetic variation within the inverted region, except for two polymorphic regions with a size of approximately 1 and 2 mb, respectively (see Supporting Information for details). Moreover, 2 mb upstream of the proximal breakpoint, the *In(3R)Mo* karyotypes were almost completely genetically invariant. We also observed a large haplotype ranging from more than 6 mb upstream to approximately 1 mb downstream of *In(3R)Mo*. In contrast to *In(3R)Mo*, the large terminal inversion (>12mb) *In(3R)C*, which spans the distal end of chromosomal arm *3R*, did not show any continuous genomic regions exhibiting highly reduced genetic variation. Nonetheless, genetic variation was locally reduced at the breakpoints of the two overlapping inversions *In(3R)Mo* and *In(3R)Payne*. The strongest reduction, showing almost complete absence of genetic variation, was found in a region of approximately 500 kb close to the distal breakpoint of *In(3R)Mo*. However, apart from locally elevated haplotype structure at the proximal breakpoint of *In(3R)C* and the telomeric part of *3R*, we did not observe elevated levels of LD (see Fig. 2B). Pairwise *F*_ST_ was increased for both inverted karyotypes within the inversions as well as in their proximity. Interestingly, we identified peaks of clear differentiation only at the proximal but not the distal breakpoints of both inversions. Moreover, despite pronounced haplotype structure in *In(3R)Mo*, we observed differences in the chromosomal distribution of elevated LD among the different arrangements, but failed to find strong variation in overall average LD (*In(3R)Mo*, LD ratio: 1.05; *In(3R)C*, LD ratio: 1.13).

### Identification of inversion-specific SNPs

**Figure 3:**
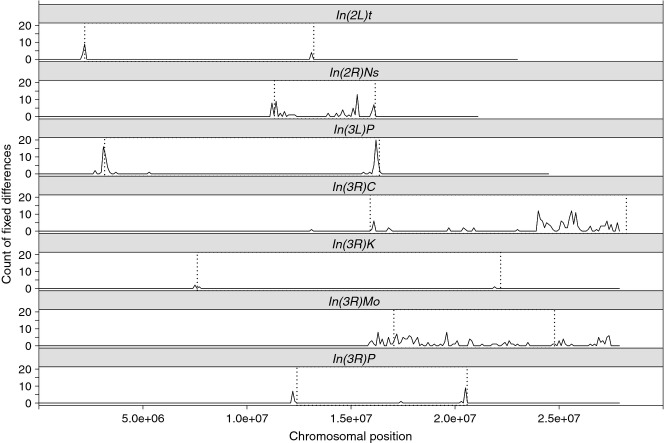
Distribution of fixed SNPs within inversions. Chromosomal distribution of inversion-specific differences based on a global sample of 167 haplotypes. The number of divergent SNPs is binned in 100-kb nonoverlapping sliding windows and plotted along the chromosomal arm carrying the corresponding inversion. Vertical dashed lines indicate the putative inversion breakpoints.

Next, we used our data to define inversion-specific SNPs that could be used as diagnostic markers for detecting and surveying seven cosmopolitan inversions including the six inversions detected in the populations of the LNS experiment (*In(2L)t, In(2R)Ns, In(3L)P*, *In(3R)Mo*, *In(3R)C* and *In(3R)Payne*) and *In(3R)K*. Alleles private to *In(2L)t*, *In(3L)P, In(3R)K* and *In(3R)Payne* were almost entirely restricted to the inversion breakpoints (Fig. 3). In contrast, alleles specific to *In(2R)Ns* and *In(3R)C* were distributed throughout these inversions (Fig. 3). For *In(3R)Mo*, we not only found marker SNPs within the inversion but also a surplus of SNPs beyond the proximal and distal breakpoints (Fig. 3). The number of marker SNPs in the different inversions varied greatly, ranging from four in *In(3R)K* to 150 in *In(3R)Mo* (Table S4). Importantly, two complementary methods for detecting false positives and a comparison of inversion frequency estimates based on karyotyping vs. marker SNPs indicated that our SNP marker set is highly reliable (Supporting Information).

### Inversion dynamics during experimental evolution

**Figure 4:**
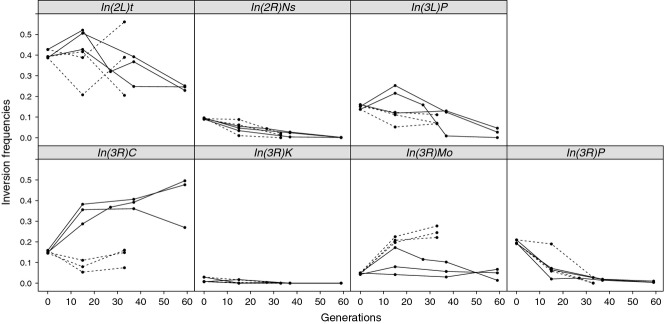
Inversion frequency trajectories during experimental evolution. Inversion frequencies estimated by marker SNPs from Pool-Seq data for the three different replicate populations in each selection regime (‘cold’ indicated by dashed and ‘hot’ indicated by solid lines) of our LNS experiment. The frequency estimates were calculated by averaging the frequencies of all marker alleles for each inversion separately.

We used these inversion-specific marker SNPs to investigate the dynamics of inversions during our experimental evolution experiment, using three replicate populations in each selection regime. For each inversion, we estimated its frequency by averaging over the frequencies of all inversion-specific SNP markers. With a baseline frequency of about 40% in the base population, *In(2L)t* was the most frequent inversion in the experiment. Its frequency fluctuated unpredictably across selection regimes and replicate populations, but the inversion remained polymorphic throughout the experiment with frequencies larger than 20% (see Fig. 4, Fig. S4A, Table S5). In contrast, *In(2R)Ns* started out at a frequency of approximately 10% in the base populations and then consistently decreased in all replicates in both selection regimes (Fig. 4, Fig. S4B, Table S5). This pattern resulted in a statistically significant difference in inversion frequency between the base population and the third time point examined in both thermal selection regimes (Table S6). Similarly, *In(3R)Payne* decreased significantly in frequency in both regimes (see Fig. 4, Fig. S4G, Table S5), a trend already noticed by Orozco-ter Wengel *et al*. (2012) for the ‘hot’ regime. Interestingly, three inversions showed a selection regime-specific behaviour. While *In(3L)P* remained stable around 15% in the ‘cold’ regime, it decreased significantly over time in the ‘hot’ regime (Fig. 4, Fig. S4C, Table S5). In contrast, *In(3R)Mo* initially segregated at a very low frequency of approximately 5% in the base populations but then consistently increased to >25% in all replicates of the ‘cold’ regime while showing inconsistent frequency patterns in the ‘hot’ regime (Fig. 4, Fig. S4F). Finally, *In(3R)C* started out at approximately 15%, then strongly increased over time in all replicates of the ‘hot’ regime, but fluctuated unpredictably in the ‘cold’ regime (Fig. 4, Fig. S4D). In good agreement with these changes in inversion frequencies as estimated from our SNP markers, we found highly significant effects of inversion type (two-way anova, *F*_5,24_ = 21.339, *P *<* *0.0001) and of the inversion type by selection regime interaction (*F*_5,24_ = 6.9793, *P *<* *0.001) in our data based on inversion frequencies observed from 275 karyotyped larvae, confirming again the reliability of our novel inversion-specific SNP markers.

### Spatial distribution of inversions in natural populations

We next used our inversion-specific SNPs to estimate inversion frequencies in two previously published Pool-Seq data sets of populations collected along latitudinal clines in North America (Fabian *et al.* 2012) and Australia (Kolaczkowski *et al.* 2011). For the North American data, we found a clinal distribution of most inversions (Fig. S5A, Table S7). *In(2L)t*, *In(3L)P* and *In(3R)Payne* showed strongly clinal patterns negatively correlated with latitude (Table S8). While *In(2L)t* and *In(3L)P* decreased linearly from south (Florida) to north (Maine), *In(3R)Payne* was very frequent (∼50%) in Florida, but almost absent in Pennsylvania and Maine (also see Fabian *et al.* 2012). In contrast, the frequencies of *In(2R)Ns, In(3R)K* and *In(3R)Mo* increased with latitude. *In(3R)C* segregated at very low frequencies and showed no clinal pattern.

Similarly, we estimated inversion frequencies for the two endpoints of the parallel but independent Australian cline (Queensland and Tasmania; cf. Kolaczkowski *et al.* 2011) (Fig. S5B, Table S7). Similar to the patterns we observed for the North American cline, we found that *In(2L)t*, *In(3L)P* and *In(3R)Payne* were much more frequent at low latitude (Queensland), but absent or at low frequency at high latitude (Tasmania). However, none of the observed frequency differences were significant according to FET (see Table S8), maybe due to the low sequence coverage in this data set. We did not detect the presence of *In(2R)Ns*, *In(3R)C, In(3R)K* and *In(3R)Mo* in the Australian data set, but due to low coverage, we were unable to determine whether these inversions occur at a very low frequency or whether they are truly absent.

## Discussion

Numerous previous studies have aimed to understand patterns of genetic variation associated with inversions in *D. melanogaster* (e.g. see Andolfatto *et al.* 2001; and references therein). Fixed genetic differences associated with inversions have been of particular interest because they may provide valuable information about the evolutionary history of these structural variants. For example, variation around inversion breakpoints has frequently been used to estimate inversion age (Hasson & Eanes 1996; Andolfatto *et al.* 1999; Matzkin *et al.* 2005). However, previous studies have been limited by the restricted amount of available data and especially by the paucity of reliable molecular markers for detecting and surveying inversions in *D. melanogaster*.

Here, we have aimed to extend these efforts using a combination of next-generation whole-genome sequence analysis and classical karyotyping of inversions in *D. melanogaster*. Specifically, by combining haplotype data from our present study (based on both individual-level sequencing and karyotyping) with publicly available haplotype information from known karyotypes in the DPGP and DPGP2 data, we have developed a new and extensive set of inversion-specific marker SNPs. These novel diagnostic markers have allowed us to characterize the frequency dynamics of seven polymorphic inversions in both laboratory and natural populations of *D. melanogaster*.

### Patterns of divergence in chromosomal inversions

Overall, we found large heterogeneity in the number and distribution of divergent SNPs for the different inversions. In three of the common large cosmopolitan inversions (*In(2L)t*, *In(3L)P* and *In(3R)Payne*) and in the rare large cosmopolitan inversion *In(3R)K,* we found only few divergent SNPs, most of which were restricted to the inversion breakpoints. These patterns agree well with previous observations for *In(2L)t* and *In(3L)P* (Andolfatto *et al.* 2001) and provide further evidence that suppression of gene flux is mainly restricted to only a few kb around the inversion breakpoints.

For *In(2R)Ns*, which is also considered to be a common cosmopolitan inversion and which has a similar age as *In(2L)t*, *In(3L)P*, *In(3R)Payne* and *In(3R)K* (Corbett-Detig & Hartl 2012), we identified fixed differences throughout the whole inversion. This inversion is markedly smaller than the other cosmopolitan inversions (∼4.8 mb), resulting in an effective recombination rate of approximately 18 cM across the inverted region (e.g. Fiston-Lavier *et al.* 2010; Comeron *et al.* 2012). As double crossing-over is unlikely to occur in regions of less than 20 cM (Navarro *et al.* 1997), presumably because the minimum distance between chiasmata is limited by crossing-over interference (McPeek & Speed 1995; Torgasheva & Borodin 2010), the pattern we have observed for *In(2R)Ns* might reflect the complete absence of double recombination and only low rates of gene conversion.

Similar to *In(2R)Ns*, we found that for two rare cosmopolitan inversions on *3R* (*In(3R)C*, *In(3R)Mo*) fixed differences were also not restricted to the breakpoint regions. *In(3R)C* is a large terminal inversion (>12 mb), and marker SNPs for this inversion showed a pronounced nonhomogeneous distribution. SNPs were found across the distal half of the inverted region, perhaps reflecting reduced recombination close to the telomere rather than an inversion-specific pattern. Alternatively, this pattern might reflect selection of coadapted *In(3R)C*-specific alleles. However, because *In(3R)C* haplotypes were only available from one population from Portugal, we cannot rule out that these patterns are highly specific.

The number and distribution of marker SNPs for *In(3R)Mo* differed markedly from all other inversions. For this inversion, we detected the highest number of marker SNPs and found them to be distributed inside the inversion as well as beyond the inversions boundaries, both proximally and distally. This strongly suggests that suppression of recombination occurs well beyond the inversion breakpoints.

### Distribution of inversions in natural populations

The pervasive clinal distribution of the cosmopolitan inversions *In(2L)t*, *In(3L)P* and *In(3R)Payne* along latitudinal gradients is well known and has been documented for numerous populations in North America, Australia and Asia already over 30 years ago (Knibb 1982). The fact that qualitatively similar frequency clines for these inversions have been observed on multiple continents has been taken as strong *prima facie* evidence for the non-neutral maintenance of these inversions by spatially varying selection. However, up-to-date, no conclusive data have been published about whether the clinal patterns for these inversions have remained stable or not. While two studies from Australia (Anderson *et al.* 1987, 2005) found that inversion clines remained stable or shifted with latitude, a study from Japan observed pronounced changes in some populations over many years (Inoue *et al.* 1984a). We were therefore interested in using our inversion-specific SNP markers to examine inversion frequencies in recently generated Pool-Seq data for the North American (Fabian *et al.* 2012) and Australian (Kolaczkowski *et al.* 2011) clines.

Despite a large difference in sequence coverage between these two recent studies (approximately 45-fold vs. 11-fold coverage), we observed clinal frequency patterns for *In(2L)t*, *In(3L)P* and *In(3R)Payne* that are in excellent qualitative agreement with previous findings from the 1970s and 1980s (Mettler *et al.* 1977; Knibb *et al.* 1981; Knibb 1982) for both the Australian and the North American cline. Remarkably, our data suggest that the inversion frequencies for *In(3R)Payne* and *In(3L)P* have remained extremely stable for more than 30 years. In contrast, for *In(2L)t,* we also observed clinal variation but detected an increase in the frequency of this inversion by approximately 20% in all populations as compared to previous observations. Although we observed strong inversion clines in the data from the Australian east coast that are qualitatively consistent with previous studies, our inversion frequency estimates for Australia were generally lower than those reported in previous work. While it is possible that these results reflect a reduction in inversion frequencies in Australia in recent years, we cannot rule out that the low sequencing coverage of the Australian data has downward-biased our estimates. Clearly, further in-depth analysis of these inversions will be necessary to understand the mechanisms that determine their dynamics and maintenance.

*In(2R)Ns*, in contrast, showed a different pattern to that observed for *In(2L)t*, *In(3L)P* and *In(3R)Payne*. Two earlier studies found this inversion to occur at a frequency of >20% in Queensland (Mettler *et al.* 1977; Knibb *et al.* 1981), but our analysis of the Australian data suggests that this inversion has either decreased to very low frequencies or that it has completely vanished in Australia. For the North American cline, we also found a pattern that contrasts with previous results: Mettler *et al*. (1977) reported that the frequency of *In(2R)Ns* decreases with increasing latitude, whereas in our analysis, this inversion showed a weakly (nonsignificant) clinal trend from approximately 0–1% frequency in Florida up to 7–10% in Maine.

The three rare cosmopolitan inversions (*In(3R)C*, *In(3R)K* and *In(3R)Mo*) were either not present in the Australian data or segregated at frequencies below our detection threshold. In contrast, for the North American east coast, we found both *In(3R)C* and *In(3R)K* to be segregating at very low frequencies, consistent with previous observations (Mettler *et al.* 1977; Knibb 1982). Surprisingly, while *In(3R)Mo* was found to be very rare and nonclinal in North America 30 years ago (Mettler *et al.* 1977), we now detect a positive correlation with latitude. This is consistent with the data of Langley *et al*. (2012) who have recently noticed a considerable increase in *In(3R)Mo* frequency (up to a frequency of approximately 18% in Raleigh, North Carolina). Together, our data indicate that *In(3R)Mo* has recently undergone a strong increase in frequency along the North American east coast. Although the reasons for this striking pattern remain unclear, the strong reduction in genetic variation within and around *In(3R)Mo* described here and in two other recent studies (Corbett-Detig & Hartl 2012; Langley *et al.* 2012) is consistent with this notion and indicates a recent origin coupled with a rapid increase in frequency.

We also found that the frequency of *In(3R)Mo* was consistently elevated in all replicates of the ‘cold’ selection regime in our LNS experiment. Strikingly, this frequency increase matched the clinal pattern observed along the North American east coast, perhaps consistent with the notion that *In(3R)Mo* is involved in cold temperature adaptation. Future work will be necessary to better understand the adaptive effect of this inversion, for example by examining the phenotypic effects of the different karyotypes.

### Implications of inversion polymorphisms for genome scans of selection

Our investigation of inversion frequency dynamics during experimental evolution clearly demonstrates that the frequencies of some inversions change consistently among replicate populations. While some inversions decreased in frequency in both thermal selection regimes, three of them changed consistently in frequency in only one of the selection regimes. A meta-analysis of inversion frequency changes during experimental evolution by Inoue (1979) has reported that inversion frequencies generally decrease during experimental evolution. However, in contrast to Inoue (1979), here we have identified two inversions (*In(3R)C* and *In(3R)Mo*) whose frequencies clearly and consistently increased over time in one of the selection regimes in our experimental evolution study. Wright–Fisher simulations of neutral evolution based on the initial inversion frequencies show that frequency changes observed for these two inversions were significantly higher than expected due to genetic drift alone (see Table S9). Thus, this pattern strongly suggests that both inversions must likely have carried one or several selection regime-specific favorable alleles. Perhaps consistent with a selective role for *In(3R)C*, this inversion has previously been found to affect bristle number variation in an artificial selection experiment (Izquierdo *et al.* 1991).

In a genome-wide analysis of our ‘hot’ selection regime, Orozco-ter Wengel *et al*. (2012) have identified the majority of candidate SNPs to be located on chromosome *3R*, which also harbors four overlapping inversions. Strikingly, two of these inversions, *In(3R)C* and *In(3R)Payne*, changed significantly in frequency in the ‘hot’ regime over the experiment. While *In(3R)C* consistently increased in all three replicate populations over time, *In(3R)Payne* decreased in frequency substantially, suggesting that this inversion is strongly selected against in our experimental evolution study. Overall, our findings are consistent with the notion that alleles associated with these inversions are major targets of selection. However, among the most significant candidate SNPs identified by Orozco-ter Wengel *et al*. (2012), only 1–3 of the marker SNPs for *In(3R)C* (depending on the data set analysed) overlapped the candidate SNPs sets. If *In(3R)C* was the only cause for the strong molecular signature of selection on *3R* in this experiment, these inversion-specific SNPs would clearly be expected to show the largest allele frequency differences, yet they do not. Instead, we hypothesize that the presence of inversions in laboratory populations can result in cryptic chromosome-specific population structure which in turn causes elevated drift and leads to a surplus of candidate SNPs. If selection is assumed to operate on top of this structure, the interpretation of the SNP data becomes very challenging. Thus, even though the inversions might play an important role in the response to selection, distinguishing the effects due to selection from those due to population structure is practically difficult. One way around this problem in experimental evolution studies using *Drosophila* to identify targets of selection would be to use inversion-free *Drosophila* species.

In natural populations, we have observed a similar phenomenon. Despite almost all sites being shared between *In(3R)Payne* and the noninverted chromosome, populations with a high *In(3R)Payne* frequency seem to harbor more variation (also see Fabian *et al.* 2012), as might be expected for a subdivided population. As inverted and noninverted chromosomes will have different allele frequencies, the contrast of populations with different inversion frequencies for the inference of selection is also challenging. On the other hand, in our previous study of clinal variation along North American cline, we found 77% of all clinal candidate SNPs to be located on *3R* and >50% of the candidates within the region spanned by *In(3R)Payne*, a highly nonrandom pattern that is consistent with spatially varying selection (Fabian *et al.* 2012) and that is also qualitatively mirrored in the Australian data (Kolaczkowski *et al.* 2011). Nonetheless, due to the difficulty of teasing apart the effects of demography and population structure vs. those of selection, we anticipate that in the future genome scans of selection might preferentially focus on chromosomes with the same karyotype status or use inversion-free systems.

In summary, the data we have reported here provide novel information on patterns of genetic variation associated with inversion karyotypes. In turn, this knowledge will facilitate future efforts in terms of characterizing genes within inversions and their effects on phenotypes.

## Conclusions

Here, we have presented a novel and robust set of molecular SNP markers for seven polymorphic chromosomal inversions in *D. melanogaster,* which will be highly useful for the analysis of Pool-Seq data in this model. Using these novel diagnostic tools, we have investigated inversion dynamics in laboratory and natural populations of *D. melanogaster*. Apart from a few recent studies that have investigated *In(3R)Payne* (Paaby *et al.* 2010), our data set is, to the best of our knowledge, the first to show that frequency clines of multiple cosmopolitan inversions (*In(2L)t, In(3L)P* and *In(3R)Payne*) have remained qualitatively stable over decades along the US east coast. Furthermore, we have identified a previously unobserved frequency cline for *In(3R)Mo,* which matches the patterns observed in our experiment evolution data. Additionally, we have observed consistent inversion frequency changes across multiple replicate populations undergoing LNS, suggesting that selective forces have shaped these patterns. While similar data already exist for the common cosmopolitan inversion *In(2L)t* (Alahiotis *et al.* 1977; Roca *et al.* 1982; Inoue *et al.* 1984b; Van Delden & Kamping 1989), this has – to our knowledge – not yet been shown for the rare cosmopolitan inversions *In(3R)C* and *In(3R)Mo*.

Although overall we have found a good correlation between our SNP-based and karyotype-based inversion frequency estimates, we would like to caution that our inference of inversion-specific SNPs is highly dependent on the available reference genomes. In particular, for *In(3R)C*, *In(3R)K* and *In(3R)Mo*, which did not occur in all populations in our combined data set, we cannot rule out that our marker SNP sets contain some false positives. Therefore, for diverged populations, inversion frequency estimates may be less accurate. Yet, given that multiple SNPs contribute to the estimates of inversion frequencies, we expect that our set of inversion-specific markers will show a reliable performance across all *Drosophila* populations.

The novel diagnostic tools we have developed here may prove powerful in future studies, especially in cases where chromosomal karyotyping is not possible, for example when adult individuals that have been caught in the wild are sequenced directly (Bergland *et al.* 2013). Our new approach may thus complement classical cytogenetic analyses, which nonetheless remain essential for unambiguously assessing all inversion polymorphisms in *D. melanogaster*.

## Notes

M.K., T.F. and C.S. conceived the study; M.K., H.v.S., B.M. and T.F. performed research; M.K., T.F. and C.S. wrote the manuscript.

## Data accessibility

BAM files containing the raw reads mapped against the *D. melanogaster* genome v. 5.18 for all single individuals are available from the European Nucleotide Archive (ENA; http://www.ebi.ac.uk/ena/) under the Accession no. PRJEB4952.

Reconstructed haploid genomes (available as FASTA files) are available from the Dryad database (http://datadryad.org) under doi:10.5061/dryad.t66 ns.

A collection of custom written Python scripts is available in the Supporting Information.

## Supporting Information

Fig S1 False-negative rates in haplotype reconstruction. **Fig. S2** Nucleotide diversity (π) and genetic differentiation (*F*_ST_) for *In(2L)t* and *In(3L)P*. **Fig. S3** Linkage disequilibrium for *In(2L)t* and *In(3L)P*. **Fig. S4** Inversion frequency trajectories during experimental evolution. **Fig. S5** Inversion frequencies in natural populations. **Fig. S6** False-positive rates in haplotype reconstruction. **Fig. S7** Patterns of recombination within *In(3R)Mo*.

Table S1 Karyotype and sex of sequenced individuals from the experimental evolution experiment. **Table S2** Individual karyotypes. **Table S3** Karyotypes from polytene chromosomes. **Table S4** Inversion-specific marker alleles. **Table S5** Inversion frequencies during the experimental evolution experiment. **Table S6** Inversion frequency differences during experimental evolution. **Table S7** Inversion frequencies in natural populations. **Table S8** Inversion frequency differences in natural populations. **Table S9** Expected inversion frequency changes due to neutral evolution. **Table S10** Reliability of inversion frequency estimates. **Table S11** Allele sharing among karyotypes. **Table S12**. Statistical power of inversion-specific marker alleles in estimating inversion frequencies.
