# Supplementary material for: Inference of chromosomal inversion dynamics from Pool-Seq data in natural and laboratory populations of Drosophila melanogaster
Source: Mol Ecol. 2013 Dec 20;23(7):1813–27. doi: 10.1111/mec.12594 (PMC4359753; doi:10.1111/mec.12594)
Supplement: Fig S1 — False-negative rates in haplotype reconstruction. Fig. S2 Nucleotide diversity (π) and genetic differentiation (FST) for In(2L)t and In(3L)P. Fig. S3 Linkage disequilibrium for In(2L)t and In(3L)P. Fig. S4 Inversion frequency trajectories during experimental evolution. Fig. S5 Inversion frequencies in natural populations. Fig. S6 False-positive rates in haplotype reconstruction. Fig. S7 Patterns of recombination within In(3R)Mo. [file mec0023-1813-SD1.zip › Supporting_software/Description of Bioinformatic Analysis Pipeline.docx]

# Description of Bioinformatics Analysis Pipeline

## (1) General remarks

This protocol describes the most important Python scripts used in the bioinformatic analyses used in this study. Most additional intermediate steps were performed using UNIX commands. The help function of each script provides a detailed description of the input and output files and the computational steps. The help function of each script can be accessed by typing the following command line: *python [program].py --help* (with [program] representing the name of the corresponding script). The help output will be printed to the terminal, starting with an example command line, followed by a description of the required parameters, and a detailed description of script usage and input/output specifications. All scripts need to be executed with Python v.2.6. Many scripts use *R* through the *rpy2* module. Thus, it is necessary to install *R* v. 2.12 and the *rpy2* Python package prior to using the analysis pipeline described below. Some of the scripts described here depend on additional modules used for parsing data produced with *PoPoolation* (Kofler *et al.* 2011a), which can be found in the modules folder. This folder needs to be located in the same directory as the main scripts. Further details are available upon request from Martin Kapun ([martin.kapun@unil.ch](mailto:martin.kapun@unil.ch))

## (2) Raw data processing and mapping

Raw reads were trimmed for base quality and mapped against the reference using *PoPoolation* (Kofler *et al.* 2011a) and *bwa* (Li & Durbin 2009) as described in the Material and Methods section. *PoPoolation2* (Kofler, Pandey, & Schlötterer 2011b) was used to synchronize pileup files into the sync file format, as described in *PoPoolation2*.

## (3) Haplotype reconstruction

As described in Materials and Methods, we used a sync file containing whole-genome sequence information of the maternal reference and 15 F1 larvae to reconstruct the paternal haplotypes using the script *extract_haplotype.py*. For further information see Materials and Methods and the help function of the script.

Example of command line:

*python extract_haplotype.py --input individuals.sync --min-coverage 20 --max-coverage 0.05 --min-count 20 --CI-mode individual --output output_file*

## (4) Calculating *π* and *F*_ST_

The consensus file output produced with *extract_haplotype.py* can be used to calculate SNP-wise *π* for two subsets of individuals and to calculate SNP-wise *F*_ST_ between the subsets using *Pi_FST4inversions.py*. See the help function of the script for further details.

Example of command line:

*python Pi_FST4inversions.py --input input.consensus --min-count 2 --data1 2,3,6,10 --data2 1,4,7 --all 0,1,2,3,4,6,7,10 --names ind1,ind2,ind3,ind4,ind4,ind5,ind7,ind8,ind11 --output output_file*

## (5) Binning results in non-overlapping windows

The script *binning_Pi-FST.py* was written to estimate average SNP-wise *π* or *F*_ST_ values in non-overlapping windows of a given size. The output can be easily parsed for visualization using *R*. See the help function of the script for further information,

Example of command line:

*python binning_Pi-FST.py --input input.pi --window-size 1000 --length inh.sam --data pi --output output_1k.pi*

## (6) Estimation of linkage disequilibrium among SNPs

Based on a consensus file generated with *extract_haplotype.py*, we used the script *LD4chromosomes.py* to calculate *r^2^* among SNPs randomly drawn along a chromosomal arm and to produce triangular heatmaps representing all pairwise comparisons. See Materials and Methods and the help function for further details.

Example of command line:

*python LD4chromosomes.py --input input.consensus --individuals 2,3,6,10 --subsample 500 --chromosome 2L --output output_2L*

## (7) Reconstruction of NJ trees based on pairwise calculations of *π*

To infer the genealogical relationship among haplotypes in specific genomic regions, we calculated pairwise *π* among all individual haplotypes used for the analyses and reconstructed neighbor-joining dendrograms using the script *Pi2tree.py.* See the help function and Materials and Methods for further details.

Example of command line:

*python Pi2tree.py --input input.consensus --individuals 2,3,6,10 --names ind2,ind3,ind6,ind10 --output output*

## (8) Conversion of DPGP data to the consensus file format

We used the script *DPGP2consensus.py* to convert the sequence information of lines from the DPGP project ([www.dpgp.org](http://www.dpgp.org)) stored in multiple FASTQ files into the consensus file format.

Example of command line:

*python DPGP2consensus.py --input_directory /dpgp_solexa_r1.0/fastq/ --output output_file.consensus*

## (9) Conversion of DPGP2 data to the consensus file format

We used the script *DPGP22consensus.py* to convert the sequence information of lines from the DPGP2 project ([www.dpgp.org](http://www.dpgp.org)) stored in a single FASTQ file into the consensus file format.

Example of command line:

*python DPGP22consensus.py --input dpgp2/dpgp2_v2_core.ID5.nohets.fastq --output output_file.consensus --length inh.sam --ID_list list.txt*

## (10) Identification of karyotype specific alleles

After combining the consensus files of all haplotype data used in the present study using UNIX commands, we identified SNPs with fixed differences for different karyotypes in the combined dataset using the script *find_fixed_alleles.py.* See the help function for further details.

Example of command line:

*python find_fixed_alleles.py --input input.consensus --data1 0,1,2,3,4 --data2 5,6,7,8 --output output.txt*

**References**

Kofler R, Orozco-terWengel P, De Maio N, Pandey RV, Nolte V *et al.* 2011a PoPoolation: a toolbox for population genetic analysis of next generation sequencing data from pooled individuals. *PLoS ONE*, **6**, e15925.

Kofler R, Pandey RV, Schlötterer C. 2011b PoPoolation2: identifying differentiation between populations using sequencing of pooled DNA samples (Pool-Seq). *Bioinformatics*, **27**, 3435–3436.

Li H, Durbin R 2009 Fast and accurate short read alignment with Burrows-Wheeler transform. *Bioinformatics*, **25**, 1754–1760.
