# Supplementary material for: Inference of chromosomal inversion dynamics from Pool-Seq data in natural and laboratory populations of Drosophila melanogaster
Source: Mol Ecol. 2013 Dec 20;23(7):1813–27. doi: 10.1111/mec.12594 (PMC4359753; doi:10.1111/mec.12594)
Supplement: Table S1 — Karyotype and sex of sequenced individuals from the experimental evolution experiment. Table S2 Individual karyotypes. Table S3 Karyotypes from polytene chromosomes. Table S4 Inversion-specific marker alleles. Table S5 Inversion frequencies during the experimental evolution experiment. Table S6 Inversion frequency differences during experimental evolution. Table S7 Inversion frequencies in natural populations. Table S8 Inversion frequency differences in natural populations. Table S9 Expected inversion frequency changes due to neutral evolution. Table S10 Reliability of inversion frequency estimates. Table S11 Allele sharing among karyotypes. Table S12. Statistical power of inversion-specific marker alleles in estimating inversion frequencies. [file mec0023-1813-SD2.doc]

# Supporting Information

Estimation of false negative and false positives during haplotype reconstruction 2

Number of false positives in inversion-specific fixed differences 3

Reliability of using inversion-specific fixed differences as inversion-specific markers in Pool-Seq data 4

Complex patterns of gene flux and genetic variation in overlapping inversions 5

References …………………………………………………………………………….6

Supporting Figures and Tables 7

Documentation of bioinformatics pipeline…………….………...………….………..49

## Estimation of false negative and false positives during haplotype reconstruction

Based on our crossing scheme for chromosomal karyotyping, we developed a novel bioinformatics pipeline to reconstruct sire (male parent) haplotypes from whole-genome-sequenced F1 larvae. As described in the Material and Methods section, we implemented several filtering and stringency thresholds to avoid wrongly typed alleles. Here we describe two methods, which were used to estimate the number of false positives and false negatives among reconstructed haplotypes. First, we sexed sequenced larvae based on cytology and sequencing data: male *Drosophila* individuals are homozygous for the *X* chromosome, which results in (i) large DNA staining intensity differences between autosomes and the *X* in preparations of polytene chromosomes and (ii) large coverage differences between autosomes and the *X* in next-generation sequencing data. With these two methods, we were able to unambiguously identify two male larvae in our dataset. In these individuals, only the maternal copy of the *X* chromosome was sequenced; thus, all SNPs detected on the *X* in these individuals represent sequencing or mapping errors. These data therefore allowed us to estimate the overall false positive rate. For individual number 136 (approximately 48-fold autosomal coverage) and individual number 100 (approximately 27-fold autosomal coverage) we detected 9 and 13 false positive SNPs respectively, translating into false positive rates of 4 x 10^-7^ and 5 x 10^-7^ along the *X* chromosome (approximately 22.4 mb long) for the parameter combinations used in the analysis. Supporting Figure 6 shows the false positive rate for four different parameter combinations for both male individuals. Second, in single individuals sequenced with next-generation sequencing allele frequencies of polymorphic SNPs are distributed around a frequency of 0.5 depending on sequencing depth. However, low coverages inflate the sampling error, which can result in the absence of polymorphic alleles. Given that we sequenced the reference strain used for the crosses, we were able to identify cases among the F1 hybrid sequences for which positions appeared to be fixed for an allele different than the reference. Assuming that the distribution of frequencies caused by sampling error is symmetrical, we were able to obtain false negative rates for our data. Supporting Figure 1 shows the average coverages and false negative rates for each individual at different minimum coverage thresholds. In summary, our results strongly suggest that the haplotype datasets used in our analysis were not affected by high false positive and false negative rates.

## Number of false positives in inversion-specific fixed differences

In our study we developed a panel of inversion-specific fixed SNP markers, obtained by analyzing karyotype-specific nucleotide variation in an alignment of 167 *D. melanogaster* genomes originating from Africa, Europe and North America (see Supporting Table 1). To rule out false positives due to sampling artifacts, we estimated false positive rates using permutations. We randomly assigned individuals as being inverted or non-inverted a 100 times (in the same proportions as in the real data) and counted the number of falsely identified candidates. None of the permuted data resulted in any false positive candidate SNPs.

We further tested whether the inversion-specific markers SNPs identified inversion frequency differences more accurately than randomly selected SNPs located within the boundaries of corresponding inversions. We therefore performed Cochran-Mantel-Haenszel (CMH) tests between the base population and consecutive experimental generations in both selection regimes for each marker SNP separately, as described in Materials and Methods. To obtain a combined result we averaged over all *χ*^2^ values. We then randomly sampled 10,000 times the same number of SNPs as the real marker SNPs and performed CMH tests; for each of these 10,000 sets we counted how often the *χ*^2^ values from the random data were larger than for the marker SNPs. By sampling from the tails of this distribution we obtained empirical *P*-value estimates, based on a cut-off defined by the *χ*^2^ value of the real marker SNPs. Under the null hypothesis, inversion-specific alleles would be expected to not perform better in predicting inversion frequencies than randomly drawn samples from within the inversion. The empirical *P*-values from this analysis are shown in Supporting Table 12. We found that our marker SNPs performed significantly better than randomly drawn SNPs for those inversions whose frequencies changed most strongly over time in our selection experiment (i.e., *In(3R)P* and *In(2R)Ns* in both regimes; *In(3R)Mo* in the “cold” regime; and *In(3R)C* in the “hot” regime), but not for inversions whose frequencies changed only weakly or which were segregating at very low baseline frequencies.

## Reliability of using inversion-specific fixed differences as inversion-specific markers in Pool-Seq data

Next, we examined the extent to which our fixed marker SNPs provide accurate estimates of inversion frequencies in our Pool-Seq data. To do so, we compared empirical data based on karyotyping of flies from our laboratory natural selection experiment with inversion frequencies estimated from our Pool-Seq data. Using Fisher’s exact tests (FET) we asked whether inversion frequency counts obtained from karyotyping differ significantly from the average inversion frequency counts as estimated by our inversion-specific SNP markers. None of the 36 tests (6 inversions  × 2 treatments × 3 replicates; Supporting Table 10) resulted in *P*-values <0.05. Therefore, our results clearly suggest that our set of inversion-specific marker SNPs is very reliable and robust in terms of accurately estimating inversion frequencies from Pool-Seq datasets.

## Complex patterns of gene flux and genetic variation in overlapping inversions

The presence of three overlapping inversions on *3R* in our haplotype data provides a unique opportunity for studying genetic exchange between different arrangements. We focused on *In(3R)Mo* which was represented by 5 chromosomes in our dataset. With the exception of two polymorphic regions within the inversion boundaries, *In(3R)Mo* showed almost complete absence of genetic variation within and beyond the inversion boundaries (see Figure 1). We identified two individuals (numbers 96 and 100) which carried polymorphisms within the inversion body of *In(3R)Mo* (see Supporting Figure 7A). To further explore the genealogical relationship among all chromosomes with different arrangements in these two polymorphic regions, we reconstructed phylogenetic trees based on *π*, using only SNPs with unique alleles in individuals 96 and/or 100 (see Supporting Figure 7A-C). Therefore, we constructed distance matrices by calculating average *π* for all possible chromosome pairs in the sample and used the neighbor-joining method to generate dendrograms using the *R* package ‘ape’ (Paradis *et al*. 2004). We determined the statistical significance of each node by bootstraping 1000 times, each time randomly drawing a subset corresponding to 10% of all SNPs from the dataset, and then calculated consensus trees using ‘ape’ in *R*.

Interestingly, in all phylogenies either one or both of these individuals differed significantly from all other *In(3R)Mo* chromosomes. Specifically, in the proximal half of the first polymorphic region, both individuals were highly similar and clustered with the standard arrangement and with the single *In(3R)Payne* individual (see Supporting Figure 7A), whereas individual 100 only clustered with the chromosome carrying *In(3R)Payne* in the distal half (see Supporting Figure 7B). In contrast, in the second region only individual 96 clustered with standard arrangement chromosomes (see Supporting Figure 7C). To further analyze the amount of allele sharing between the different arrangements, we extracted SNPs specific to both individuals and counted how often these alleles segregated in other arrangements. Remarkably, the alleles specific to individual 96 were entirely shared with the standard arrangement but not associated with a single haplotype. Similarly, the majority of alleles (>75 %) specific to individual 100 from the first region were also shared with the standard arrangement. A major proportion of the alleles specific to both individuals was also shared with *In(3R)C* and with the single individual carrying *In(3R)Payne* (see Supporting Table 11)*.* In summary, these findings indicate that the patterns observed within *In(3R)Mo* haplotypes are the result of multiple recent recombination events, at first between different arrangements and subsequently between *In(3R)Mo* haplotypes.

**References**

Paradis E., Claude J. and K. Strimmer, 2004 APE: Analyses of Phylogenetics and Evolution in R language. Bioinformatics **20**: 289–290.

## Supporting Figures and Tables

**Supporting Figure 6. False negative rates in haplotype reconstruction.** Average coverages based on next-generation sequencing data for the reference strain and all 15 F1 hybrids (grey line) and false negative rate estimates for different minimum coverage thresholds for each individual separately. See Supporting Text for further details.

**Supporting Figure 2. Nucleotide diversiy (*π*) and genetic differentiation (*F*_ST_) for *In(2L)t* and *In(3L)P*.** Line plots showing *π* averaged in 100-kb non-overlapping sliding windows of individuals with standard (blue) and inverted (red) chromosomal arrangement; *F*_ST_ values (black) show the amount of genetic differentiation between these arrangements. (A) results for *In(2L)t*, for five individuals of each karyotype. (B) results for *In(3L)P*, for six individuals of each karyotype. In both (A) and (B), the black lines represent the putative boundaries of the corresponding inversions.

**Supporting Figure 3. Linkage disequilibrium for *In(2L)t* and *In(3L)P*.** Triangular heatmaps showing the values of pairwise calculations of *r*^2^ for 5000 randomly sampled SNPs across each chromosome. The bottom half shows the results for individuals with the inverted arrangement, whereas the top half shows the results for standard arrangement chromosomes, based on the same number of individuals as for the inverted karyotype. The chromosomal location of each inversion is highlighted as a red line. (A) Plots for *2L*, with *In(2L)t* at the bottom and the standard arrangement at the top (based on 5 individuals). (B) Plots for *3L*, with *In(3L)P* at the bottom and the standard arrangement at the top (based on 4 individuals).

**Supporting Figure 4. Inversion frequency trajectories during experimental evolution.** Box plots showing the allele frequency distributions of inversion-specific SNP markers across different selection regimes (rows; “hot” and “cold”) and replicate populations (columns) in our laboratory natural selection experiment. We used the median of each distribution to estimate inversion frequencies. (A) Results for *In(2L)t*; (B) for *In(2R)Ns*; (C) for *In(3L)P*; (D) for *In(3R)C*; (E) for *In(3R)K*; (F) for *In(3R)Mo* and (G) for *In(3R)Payne.* We performed CMH tests to test for significant frequency differences between generation 0 and consecutive generations in the experimental evolution experiment for each candidate SNP separately. Combined results were obtained by averaging across all *P*-values of all marker SNPs. Green stars indicate significant results between the base population (generation 0) and the corresponding evolved populations at subsequent timepoints during the selection experiment (* *P*<0.05, ** *P* <0.01, *** *P* <0.001).

**Supporting Figure 5. Inversion frequencies in natural populations.** Box plots showing allele frequencies of inversion specific SNP markers in latitudinal populations from Australia (A; Kolaczkowski *et al*. 2011) and North America (B; Fabian *et al.* 2012). We performed Fisher’s Exact tests (FET) to test for significant frequency differences between the population at the lowest latitude (i.e., Florida and Queensland, respectively) and all other populations along each cline for each candidate SNP separately. Combined results were obtained by averaging across all *P*-values of all marker SNPs. Green stars indicate significant results for the comparison between the lowest-latitude population and the other populations (* *P*<0.05, ** *P*<0.01, *** *P*< 0.001).

**Supporting Figure 6. False positive rates in haplotype reconstruction*.*** False positive rates estimated for two male F1 hybrids (individuals 100 and 136) for different filtering parameters (minimum allele count and minimum mapping quality), as described in Materials and Methods; also see Supporting Text for further details.

**Supporting Figure 7. Patterns of recombination within *In(3R)Mo.*** The center plot shows *π *averaged in 100-kb non-overlapping sliding windows for three different combinations of individuals carrying *In(3R)Mo* within the inverted region on *3R*. The orange line represents individuals 80, 129 and 150; the black line the three former individuals plus individual 100; and the grey line individuals 80,129, 150 and 96. Dendrograms were generated from distance matrices based on *π *calculated for all pairwise comparisons using SNPs with unique alleles in individuals 96 or 100. The chromosomal arrangements of individuals in the trees are color-coded, with *In(3R)Mo* shown in red, *In(3R)C* in green, *In(3R)Payne* in blue and the standard arrangement in black. We used bootstrapping to test for the consistency of the tree topologies. Branches with >95% bootstrapping support are indicated with a purple dot. Trees in (A) and (B) are based on SNPs specific for individual 96, whereas (C) is based on SNPs with unique alleles in individual 100. The length of the scale bar in each plot corresponds to *π  =* 0.1.

**Supporting Figure 1**

**Supporting Figure 2**

**Supporting Figure 3**


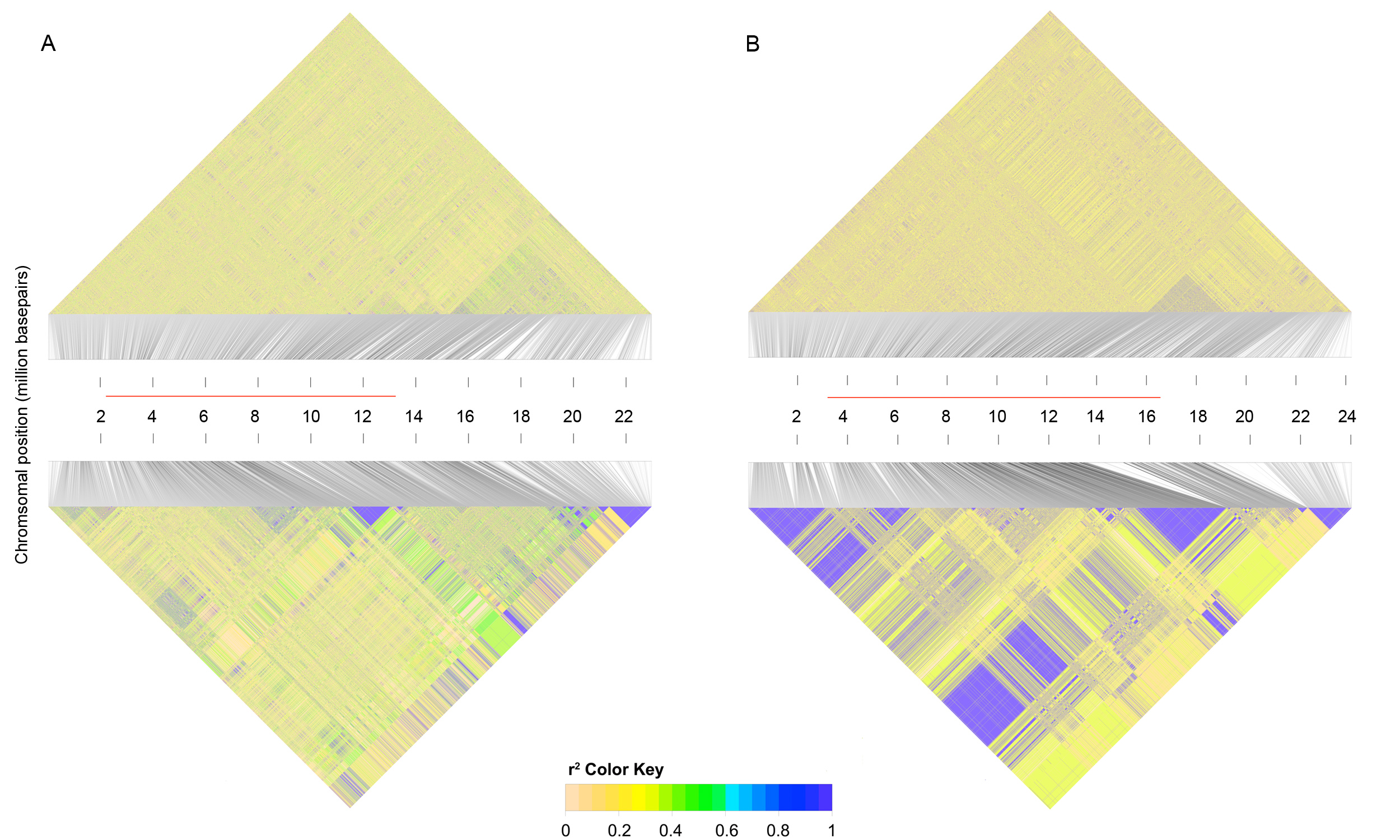


**Supporting Figure 4**

**A**

**B**

**C**

**D**

**E**

**F**

**G**

**Supporting Figure 5**

**A**

**B**

**Supporting Figure 6**

**Supporting Figure 7**

Supporting Table 1. Karyotype and sex of sequenced individuals from the experimental evolution experiment. Number of individual (ID), selection regime (“hot”, “cold”; replicates (R) 1-3), karyotype and sex of the 15 individuals sequenced from the experimental evolution experiment. Also see Materials and Methods.

| **ID** | **Regime** | ***In(2L)t*** | ***In(2R)Ns*** | ***In(3L)P*** | ***In(3R)C*** | ***In(3R)Mo*** | ***In(3R)P*** | **Sex** |
| --- | --- | --- | --- | --- | --- | --- | --- | --- |
| 21 | cold-R3 | 0 | 1 | 0 | 0 | 0 | 0 | f |
| 52 | cold-R2 | 0 | 0 | 0 | 1 | 0 | 0 | f |
| 53 | cold-R2 | 0 | 0 | 0 | 1 | 0 | 0 | f |
| 80 | cold-R2 | 1 | 0 | 0 | 0 | 1 | 0 | f |
| 89 | cold-R2 | 0 | 0 | 1 | 0 | 0 | 0 | f |
| 91 | cold-R1 | 0 | 0 | 1 | 0 | 0 | 0 | f |
| 96 | cold-R1 | 0 | 0 | 0 | 0 | 1 | 0 | f |
| 100 | cold-R1 | 1 | 0 | 0 | 0 | 1 | 0 | m |
| 106 | hot-R1 | 1 | 0 | 0 | 1 | 0 | 0 | f |
| 117 | hot-R1 | 0 | 0 | 1 | 1 | 0 | 0 | f |
| 129 | hot-R1 | 0 | 0 | 0 | 0 | 1 | 0 | f |
| 136 | hot-R1 | 1 | 0 | 0 | 1 | 0 | 0 | m |
| 143 | hot-R2 | 1 | 0 | 1 | 1 | 0 | 0 | f |
| 150 | hot-R2 | 0 | 0 | 0 | 0 | 1 | 0 | f |
| 168 | hot-R2 | 0 | 0 | 0 | 0 | 0 | 1 | f |

**Supporting Table 2.** **Individual karyotypes.**  Data source, geographic origin, individual number (ID) and karyotype for all 167 individuals used to identify fixed differences between chromosomal arrangements.

| **Source** | **Origin** | **ID** | ***In(2L)t*** | ***In(2R)Ns*** | ***In(3L)P*** | ***In(3R)C*** | ***In(3R)K*** | ***In(3R)Mo*** | ***In(3R)P*** |
| --- | --- | --- | --- | --- | --- | --- | --- | --- | --- |
| this study | Europe | 21 | 0 | 1 | 0 | 0 | 0 | 0 | 0 |
| this study | Europe | 52 | 0 | 0 | 0 | 1 | 0 | 0 | 0 |
| this study | Europe | 53 | 0 | 0 | 0 | 1 | 0 | 0 | 0 |
| this study | Europe | 80 | 1 | 0 | 0 | 0 | 0 | 1 | 0 |
| this study | Europe | 89 | 0 | 0 | 1 | 0 | 0 | 0 | 0 |
| this study | Europe | 91 | 0 | 0 | 1 | 0 | 0 | 0 | 0 |
| this study | Europe | 96 | 0 | 0 | 0 | 0 | 0 | 1 | 0 |
| this study | Europe | 100 | 1 | 0 | 0 | 0 | 0 | 1 | 0 |
| this study | Europe | 106 | 1 | 0 | 0 | 1 | 0 | 0 | 0 |
| this study | Europe | 117 | 0 | 0 | 1 | 1 | 0 | 0 | 0 |
| this study | Europe | 129 | 0 | 0 | 0 | 0 | 0 | 1 | 0 |
| this study | Europe | 136 | 1 | 0 | 0 | 1 | 0 | 0 | 0 |
| this study | Europe | 143 | 1 | 0 | 1 | 1 | 0 | 0 | 0 |
| this study | Europe | 150 | 0 | 0 | 0 | 0 | 0 | 1 | 0 |
| this study | Europe | 168 | 0 | 0 | 0 | 0 | 0 | 0 | 1 |
| DPGP2 | Africa | CK1 | 0 | 0 | 0 | 0 | 0 | 0 | 1 |
| DPGP2 | Africa | CK2 | 0 | 0 | 0 | 0 | 0 | 0 | 0 |
| DPGP2 | Africa | CO1 | 0 | 0 | 0 | 0 | 1 | 0 | 0 |
| DPGP2 | Africa | CO10N | 0 | 0 | 0 | 0 | 1 | 0 | 0 |
| DPGP2 | Africa | CO13N | 0 | 0 | 0 | 0 | 1 | 0 | 0 |
| DPGP2 | Africa | CO14 | 1 | 0 | 0 | 0 | 0 | 0 | 1 |
| DPGP2 | Africa | CO15N | 0 | 0 | 0 | 0 | 1 | 0 | 0 |
| DPGP2 | Africa | CO16 | 0 | 0 | 0 | 0 | 1 | 0 | 0 |
| DPGP2 | Africa | CO2 | 0 | 0 | 0 | 0 | 1 | 0 | 0 |
| DPGP2 | Africa | CO4N | 0 | 0 | 0 | 0 | 1 | 0 | 0 |
| DPGP2 | Africa | CO8N | 0 | 0 | 0 | 0 | 1 | 0 | 0 |
| DPGP2 | Africa | CO9N | 0 | 0 | 0 | 0 | 1 | 0 | 0 |
| DPGP2 | Africa | ED10N | 0 | 0 | 0 | 0 | 0 | 0 | 0 |
| DPGP2 | Africa | ED2 | 0 | 0 | 0 | 0 | 0 | 0 | 0 |
| DPGP2 | Africa | ED3 | 0 | 0 | 0 | 0 | 0 | 0 | 0 |
| DPGP2 | Africa | ED5N | 0 | 0 | 0 | 0 | 0 | 0 | 0 |
| DPGP2 | Africa | ED6N | 0 | 0 | 0 | 0 | 0 | 0 | 0 |
| DPGP2 | Africa | EZ2 | 1 | 0 | 0 | 0 | 0 | 0 | 0 |
| DPGP2 | Africa | EZ25 | 1 | 0 | 0 | 0 | 0 | 0 | 0 |
| DPGP2 | Africa | EZ5N | 0 | 0 | 0 | 0 | 0 | 0 | 0 |
| DPGP2 | Africa | EZ9N | 1 | 0 | 0 | 0 | 0 | 0 | 0 |
| DPGP2 | Europe | FR14 | 0 | 0 | 0 | 0 | 0 | 0 | 0 |
| DPGP2 | Europe | FR151 | 0 | 0 | 0 | 0 | 0 | 0 | 0 |
| DPGP2 | Europe | FR180 | 1 | 0 | 0 | 0 | 0 | 0 | 1 |
| DPGP2 | Europe | FR217 | 0 | 0 | 1 | 0 | 1 | 0 | 0 |
| DPGP2 | Europe | FR229 | 0 | 0 | 0 | 0 | 0 | 0 | 1 |
| DPGP2 | Europe | FR310 | 0 | 0 | 0 | 0 | 0 | 1 | 0 |
| DPGP2 | Europe | FR361 | 0 | 0 | 1 | 0 | 0 | 0 | 1 |
| DPGP2 | Europe | FR70 | 0 | 0 | 0 | 0 | 0 | 0 | 0 |
| DPGP2 | Africa | GA125 | 1 | 0 | 0 | 0 | 1 | 0 | 0 |
| DPGP2 | Africa | GA129 | 1 | 0 | 0 | 0 | 0 | 0 | 0 |
| DPGP2 | Africa | GA130 | 0 | 0 | 0 | 0 | 0 | 0 | 0 |
| DPGP2 | Africa | GA132 | 0 | 0 | 0 | 0 | 0 | 0 | 1 |
| DPGP2 | Africa | GA141 | 1 | 0 | 0 | 0 | 0 | 0 | 0 |
| DPGP2 | Africa | GA145 | 1 | 0 | 0 | 0 | 0 | 0 | 0 |
| DPGP2 | Africa | GA160 | 1 | 0 | 0 | 0 | 0 | 0 | 1 |
| DPGP2 | Africa | GA185 | 0 | 0 | 0 | 0 | 0 | 0 | 1 |
| DPGP2 | Africa | GA191 | 0 | 0 | 0 | 0 | 0 | 0 | 0 |
| DPGP2 | Africa | GU10 | 0 | 0 | 0 | 0 | 1 | 0 | 0 |
| DPGP2 | Africa | GU2 | 0 | 0 | 0 | 0 | 0 | 0 | 0 |
| DPGP2 | Africa | GU6 | 0 | 0 | 0 | 0 | 1 | 0 | 0 |
| DPGP2 | Africa | GU7 | 1 | 0 | 0 | 0 | 0 | 0 | 1 |
| DPGP2 | Africa | GU9 | 0 | 0 | 0 | 0 | 0 | 0 | 1 |
| DPGP2 | Africa | KN133N | 1 | 0 | 0 | 0 | 0 | 0 | 0 |
| DPGP2 | Africa | KN20N | 0 | 0 | 0 | 0 | 1 | 0 | 0 |
| DPGP2 | Africa | KN34 | 0 | 0 | 0 | 0 | 0 | 0 | 0 |
| DPGP2 | Africa | KN35 | 1 | 0 | 0 | 0 | 0 | 0 | 0 |
| DPGP2 | Africa | KN6 | 0 | 0 | 0 | 0 | 0 | 0 | 0 |
| DPGP2 | Africa | KR39 | 1 | 0 | 0 | 0 | 0 | 0 | 0 |
| DPGP2 | Africa | KR42 | 1 | 1 | 0 | 0 | 0 | 0 | 0 |
| DPGP2 | Africa | KR4N | 1 | 0 | 0 | 0 | 0 | 0 | 0 |
| DPGP2 | Africa | KR7 | 1 | 0 | 0 | 0 | 0 | 0 | 0 |
| DPGP2 | Africa | KT1 | 0 | 0 | 0 | 0 | 0 | 0 | 0 |
| DPGP2 | Africa | KT6 | 0 | 1 | 0 | 0 | 0 | 0 | 0 |
| DPGP2 | Africa | NG10N | 1 | 0 | 1 | 0 | 0 | 0 | 1 |
| DPGP2 | Africa | NG1N | 1 | 1 | 0 | 0 | 1 | 0 | 0 |
| DPGP2 | Africa | NG3N | 1 | 0 | 1 | 0 | 0 | 0 | 0 |
| DPGP2 | Africa | NG6N | 0 | 0 | 0 | 0 | 0 | 0 | 0 |
| DPGP2 | Africa | NG7 | 0 | 0 | 0 | 0 | 0 | 0 | 0 |
| DPGP2 | Africa | NG9 | 1 | 1 | 1 | 0 | 0 | 0 | 0 |
| DPGP2 | Africa | RC1 | 0 | 0 | 0 | 0 | 0 | 0 | 0 |
| DPGP2 | Africa | RC5 | 0 | 0 | 0 | 0 | 0 | 0 | 0 |
| DPGP2 | Africa | RG10 | 0 | 0 | 0 | 0 | 0 | 0 | 0 |
| DPGP2 | Africa | RG11N | 0 | 0 | 0 | 0 | 0 | 0 | 0 |
| DPGP2 | Africa | RG13N | 0 | 0 | 0 | 0 | 0 | 0 | 0 |
| DPGP2 | Africa | RG15 | 0 | 0 | 0 | 0 | 0 | 0 | 0 |
| DPGP2 | Africa | RG18N | 0 | 0 | 0 | 0 | 0 | 0 | 1 |
| DPGP2 | Africa | RG19 | 0 | 0 | 0 | 0 | 0 | 0 | 0 |
| DPGP2 | Africa | RG2 | 0 | 0 | 0 | 0 | 0 | 0 | 0 |
| DPGP2 | Africa | RG21N | 0 | 0 | 0 | 0 | 0 | 0 | 0 |
| DPGP2 | Africa | RG22 | 0 | 0 | 0 | 0 | 0 | 0 | 0 |
| DPGP2 | Africa | RG24 | 0 | 0 | 0 | 0 | 0 | 0 | 0 |
| DPGP2 | Africa | RG25 | 0 | 0 | 0 | 0 | 0 | 0 | 1 |
| DPGP2 | Africa | RG28 | 0 | 0 | 0 | 0 | 0 | 0 | 0 |
| DPGP2 | Africa | RG3 | 1 | 1 | 0 | 0 | 0 | 0 | 0 |
| DPGP2 | Africa | RG32N | 0 | 0 | 0 | 0 | 0 | 0 | 0 |
| DPGP2 | Africa | RG33 | 0 | 0 | 0 | 0 | 0 | 0 | 0 |
| DPGP2 | Africa | RG34 | 0 | 0 | 0 | 0 | 0 | 0 | 0 |
| DPGP2 | Africa | RG35 | 0 | 0 | 0 | 0 | 0 | 0 | 0 |
| DPGP2 | Africa | RG36 | 1 | 0 | 0 | 0 | 0 | 0 | 0 |
| DPGP2 | Africa | RG37N | 1 | 0 | 0 | 0 | 0 | 0 | 0 |
| DPGP2 | Africa | RG38N | 0 | 0 | 0 | 0 | 0 | 0 | 0 |
| DPGP2 | Africa | RG39 | 0 | 0 | 0 | 0 | 0 | 0 | 0 |
| DPGP2 | Africa | RG4N | 0 | 0 | 0 | 0 | 0 | 0 | 0 |
| DPGP2 | Africa | RG5 | 0 | 0 | 0 | 0 | 0 | 0 | 1 |
| DPGP2 | Africa | RG6N | 0 | 0 | 0 | 0 | 0 | 0 | 0 |
| DPGP2 | Africa | RG7 | 0 | 0 | 0 | 0 | 0 | 0 | 0 |
| DPGP2 | Africa | RG8 | 0 | 0 | 0 | 0 | 0 | 0 | 0 |
| DPGP2 | Africa | RG9 | 0 | 0 | 0 | 0 | 0 | 0 | 1 |
| DPGP2 | Africa | SP173 | 0 | 0 | 0 | 0 | 0 | 0 | 0 |
| DPGP2 | Africa | SP188 | 0 | 0 | 0 | 0 | 0 | 0 | 0 |
| DPGP2 | Africa | SP221 | 1 | 1 | 0 | 0 | 0 | 0 | 0 |
| DPGP2 | Africa | SP235 | 0 | 0 | 0 | 0 | 0 | 0 | 0 |
| DPGP2 | Africa | SP241 | 0 | 0 | 0 | 0 | 0 | 0 | 0 |
| DPGP2 | Africa | SP254 | 0 | 0 | 0 | 0 | 0 | 0 | 0 |
| DPGP2 | Africa | SP80 | 0 | 0 | 0 | 0 | 0 | 0 | 0 |
| DPGP2 | Africa | TZ10 | 1 | 0 | 0 | 0 | 0 | 0 | 0 |
| DPGP2 | Africa | TZ14 | 1 | 0 | 0 | 0 | 1 | 0 | 0 |
| DPGP2 | Africa | TZ8 | 1 | 0 | 0 | 0 | 0 | 0 | 0 |
| DPGP2 | Africa | UG19 | 0 | 0 | 0 | 0 | 0 | 0 | 0 |
| DPGP2 | Africa | UG28N | 0 | 0 | 0 | 0 | 0 | 0 | 0 |
| DPGP2 | Africa | UG5N | 0 | 0 | 0 | 0 | 0 | 0 | 0 |
| DPGP2 | Africa | UG7 | 0 | 0 | 0 | 0 | 0 | 0 | 0 |
| DPGP2 | Africa | UM118 | 1 | 0 | 0 | 0 | 0 | 0 | 0 |
| DPGP2 | Africa | UM37 | 0 | 0 | 0 | 0 | 1 | 0 | 0 |
| DPGP2 | Africa | UM526 | 1 | 0 | 0 | 0 | 0 | 0 | 0 |
| DPGP2 | Africa | ZI261 | 0 | 0 | 0 | 0 | 0 | 0 | 0 |
| DPGP2 | Africa | ZI268 | 0 | 0 | 0 | 0 | 0 | 0 | 0 |
| DPGP2 | Africa | ZI468 | 0 | 0 | 0 | 0 | 0 | 0 | 0 |
| DPGP2 | Africa | ZI91 | 0 | 0 | 0 | 0 | 0 | 0 | 0 |
| DPGP2 | Africa | ZL130 | 0 | 0 | 0 | 0 | 0 | 0 | 0 |
| DPGP2 | Africa | ZO65 | 1 | 1 | 0 | 0 | 0 | 0 | 0 |
| DPGP2 | Africa | ZS11 | 1 | 0 | 0 | 0 | 0 | 0 | 0 |
| DPGP2 | Africa | ZS37 | 0 | 0 | 0 | 0 | 1 | 0 | 0 |
| DPGP2 | Africa | ZS5 | 0 | 1 | 0 | 0 | 0 | 0 | 0 |
| DPGP | North America | RAL-301 | 1 | 0 | 0 | 0 | 0 | 0 | 0 |
| DPGP | North America | RAL-303 | 0 | 0 | 0 | 0 | 0 | 0 | 0 |
| DPGP | North America | RAL-304 | 0 | 1 | 0 | 0 | 0 | 0 | 0 |
| DPGP | North America | RAL-306 | 0 | 0 | 0 | 0 | 0 | 0 | 0 |
| DPGP | North America | RAL-307 | 0 | 0 | 0 | 0 | 0 | 0 | 0 |
| DPGP | North America | RAL-313 | 1 | 0 | 0 | 0 | 0 | 0 | 0 |
| DPGP | North America | RAL-315 | 0 | 0 | 0 | 0 | 0 | 0 | 0 |
| DPGP | North America | RAL-324 | 0 | 0 | 0 | 0 | 0 | 1 | 0 |
| DPGP | North America | RAL-335 | 0 | 0 | 0 | 0 | 0 | 0 | 0 |
| DPGP | North America | RAL-357 | 0 | 0 | 0 | 0 | 0 | 0 | 0 |
| DPGP | North America | RAL-358 | 1 | 0 | 0 | 0 | 0 | 1 | 0 |
| DPGP | North America | RAL-360 | 0 | 0 | 0 | 0 | 0 | 0 | 0 |
| DPGP | North America | RAL-362 | 0 | 0 | 0 | 0 | 0 | 0 | 0 |
| DPGP | North America | RAL-365 | 0 | 0 | 0 | 0 | 0 | 0 | 0 |
| DPGP | North America | RAL-375 | 0 | 0 | 0 | 0 | 0 | 0 | 0 |
| DPGP | North America | RAL-379 | 0 | 0 | 0 | 0 | 0 | 0 | 0 |
| DPGP | North America | RAL-380 | 0 | 0 | 0 | 0 | 0 | 0 | 0 |
| DPGP | North America | RAL-391 | 0 | 0 | 0 | 0 | 0 | 0 | 0 |
| DPGP | North America | RAL-399 | 0 | 0 | 0 | 0 | 0 | 0 | 0 |
| DPGP | North America | RAL-427 | 0 | 0 | 0 | 0 | 0 | 0 | 0 |
| DPGP | North America | RAL-437 | 0 | 0 | 0 | 0 | 0 | 1 | 0 |
| DPGP | North America | RAL-486 | 0 | 0 | 0 | 0 | 0 | 0 | 0 |
| DPGP | North America | RAL-514 | 0 | 0 | 0 | 0 | 0 | 0 | 0 |
| DPGP | North America | RAL-517 | 0 | 0 | 0 | 0 | 0 | 0 | 0 |
| DPGP | North America | RAL-555 | 0 | 0 | 0 | 0 | 0 | 1 | 0 |
| DPGP | North America | RAL-639 | 0 | 0 | 0 | 0 | 0 | 0 | 0 |
| DPGP | North America | RAL-705 | 0 | 0 | 0 | 0 | 0 | 0 | 0 |
| DPGP | North America | RAL-707 | 0 | 0 | 0 | 0 | 0 | 1 | 0 |
| DPGP | North America | RAL-714 | 0 | 0 | 0 | 0 | 0 | 1 | 0 |
| DPGP | North America | RAL-730 | 0 | 0 | 0 | 0 | 0 | 0 | 0 |
| DPGP | North America | RAL-732 | 0 | 0 | 0 | 0 | 1 | 0 | 0 |
| DPGP | North America | RAL-765 | 0 | 0 | 0 | 0 | 0 | 0 | 0 |
| DPGP | North America | RAL-774 | 0 | 0 | 0 | 0 | 0 | 0 | 0 |
| DPGP | North America | RAL-786 | 0 | 0 | 0 | 0 | 0 | 0 | 1 |
| DPGP | North America | RAL-799 | 0 | 0 | 0 | 0 | 0 | 0 | 0 |
| DPGP | North America | RAL-820 | 0 | 0 | 0 | 0 | 0 | 1 | 0 |
| DPGP | North America | RAL-852 | 0 | 1 | 0 | 0 | 0 | 0 | 0 |

**Supporting Table 3.** **Karyotypes from polytene chromosomes.**  Total number of chromosomes sampled (n) and number of inverted chromosomes identified per generation, treatment and replicate in the laboratory natural selection experiment. Note that for the Base population, we picked single males from randomly drawn isofemale lines (which were initially used to establish the starting population. In contrast, we randomly drew males directly from the selected populations. In both cases males were used for crosses with the non-inverted reference strain (*y*[1]; *cn*[1] *bw*[1] *sp*[1]).

| **Generation** | **Treatment** | **Replicate** | **n** | ***In(2L)t*** | ***In(2R)Ns*** | ***In(3L)P*** | ***In(3R)C*** | ***In(3R)Mo*** | ***In(3R)P*** |
| --- | --- | --- | --- | --- | --- | --- | --- | --- | --- |
| Base |  |  | 37 | 12 | 2 | 1 | 5 | 4 | 4 |
| 34 | cold | 1 | 36 | 13 | 0 | 3 | 2 | 7 | 3 |
| 34 | cold | 2 | 45 | 4 | 0 | 2 | 12 | 12 | 0 |
| 34 | cold | 3 | 30 | 10 | 2 | 0 | 3 | 6 | 0 |
| 60 | hot | 1 | 42 | 15 | 0 | 2 | 19 | 2 | 0 |
| 60 | hot | 2 | 44 | 10 | 0 | 3 | 15 | 1 | 2 |
| 60 | hot | 3 | 41 | 16 | 0 | 0 | 17 | 1 | 0 |

Supporting Table 4. Inversion-specific marker alleles. Chromosomal position and inversion-specific allele for the fixed differences between the corresponding inversion and all other chromosomal arrangements, based on 167 chromosomes.

| **Inversion** | **Chromosome** | **Position** | **Allele** |
| --- | --- | --- | --- |
| *In(2L)t* | *2L* | 2166548 | A |
| *In(2L)t* | *2L* | 2166622 | G |
| *In(2L)t* | *2L* | 2166626 | A |
| *In(2L)t* | *2L* | 2204678 | A |
| *In(2L)t* | *2L* | 2209048 | C |
| *In(2L)t* | *2L* | 2214322 | T |
| *In(2L)t* | *2L* | 2225369 | T |
| *In(2L)t* | *2L* | 2226971 | G |
| *In(2L)t* | *2L* | 2233906 | A |
| *In(2L)t* | *2L* | 2234101 | A |
| *In(2L)t* | *2L* | 2246686 | T |
| *In(2L)t* | *2L* | 2255218 | A |
| *In(2L)t* | *2L* | 13139098 | C |
| *In(2L)t* | *2L* | 13155257 | T |
| *In(2L)t* | *2L* | 13172139 | T |
| *In(2L)t* | *2L* | 13186585 | A |
| *In(2R)Ns* | *2R* | 11279637 | A |
| *In(2R)Ns* | *2R* | 11291326 | A |
| *In(2R)Ns* | *2R* | 11291656 | A |
| *In(2R)Ns* | *2R* | 11294553 | A |
| *In(2R)Ns* | *2R* | 11295105 | A |
| *In(2R)Ns* | *2R* | 11295408 | A |
| *In(2R)Ns* | *2R* | 11297771 | T |
| *In(2R)Ns* | *2R* | 11298425 | C |
| *In(2R)Ns* | *2R* | 11363601 | T |
| *In(2R)Ns* | *2R* | 11416627 | T |
| *In(2R)Ns* | *2R* | 11416743 | G |
| *In(2R)Ns* | *2R* | 11428502 | G |
| *In(2R)Ns* | *2R* | 11452011 | C |
| *In(2R)Ns* | *2R* | 11453509 | T |
| *In(2R)Ns* | *2R* | 11459978 | G |
| *In(2R)Ns* | *2R* | 11467228 | T |
| *In(2R)Ns* | *2R* | 11470424 | T |
| *In(2R)Ns* | *2R* | 11471637 | T |
| *In(2R)Ns* | *2R* | 11620344 | A |
| *In(2R)Ns* | *2R* | 11685989 | T |
| *In(2R)Ns* | *2R* | 11817613 | A |
| *In(2R)Ns* | *2R* | 11818383 | T |
| *In(2R)Ns* | *2R* | 11826149 | T |
| *In(2R)Ns* | *2R* | 12007749 | A |
| *In(2R)Ns* | *2R* | 12154859 | A |
| *In(2R)Ns* | *2R* | 12250521 | T |
| *In(2R)Ns* | *2R* | 12394846 | G |
| *In(2R)Ns* | *2R* | 13942780 | A |
| *In(2R)Ns* | *2R* | 13944397 | C |
| *In(2R)Ns* | *2R* | 14352759 | A |
| *In(2R)Ns* | *2R* | 14362949 | T |
| *In(2R)Ns* | *2R* | 14582447 | T |
| *In(2R)Ns* | *2R* | 14633978 | T |
| *In(2R)Ns* | *2R* | 14641278 | A |
| *In(2R)Ns* | *2R* | 14672926 | A |
| *In(2R)Ns* | *2R* | 14674348 | T |
| *In(2R)Ns* | *2R* | 14735385 | G |
| *In(2R)Ns* | *2R* | 14995376 | T |
| *In(2R)Ns* | *2R* | 15117841 | T |
| *In(2R)Ns* | *2R* | 15122558 | G |
| *In(2R)Ns* | *2R* | 15124138 | T |
| *In(2R)Ns* | *2R* | 15154801 | A |
| *In(2R)Ns* | *2R* | 15160191 | A |
| *In(2R)Ns* | *2R* | 15289938 | G |
| *In(2R)Ns* | *2R* | 15303213 | T |
| *In(2R)Ns* | *2R* | 15303225 | A |
| *In(2R)Ns* | *2R* | 15335793 | T |
| *In(2R)Ns* | *2R* | 15339141 | T |
| *In(2R)Ns* | *2R* | 15339337 | T |
| *In(2R)Ns* | *2R* | 15344384 | A |
| *In(2R)Ns* | *2R* | 15345300 | T |
| *In(2R)Ns* | *2R* | 15348825 | A |
| *In(2R)Ns* | *2R* | 15364662 | C |
| *In(2R)Ns* | *2R* | 15364670 | C |
| *In(2R)Ns* | *2R* | 15366984 | A |
| *In(2R)Ns* | *2R* | 15367369 | A |
| *In(2R)Ns* | *2R* | 15370164 | A |
| *In(2R)Ns* | *2R* | 16023748 | T |
| *In(2R)Ns* | *2R* | 16071561 | T |
| *In(2R)Ns* | *2R* | 16073117 | T |
| *In(2R)Ns* | *2R* | 16100012 | T |
| *In(2R)Ns* | *2R* | 16116600 | A |
| *In(2R)Ns* | *2R* | 16117724 | T |
| *In(2R)Ns* | *2R* | 16152311 | C |
| *In(2R)Ns* | *2R* | 16152687 | G |
| *In(2R)Ns* | *2R* | 16160042 | T |
| *In(2R)Ns* | *2R* | 16163328 | T |
| *In(3L)P* | *3L* | 2759715 | C |
| *In(3L)P* | *3L* | 2760784 | T |
| *In(3L)P* | *3L* | 3054925 | C |
| *In(3L)P* | *3L* | 3133022 | G |
| *In(3L)P* | *3L* | 3135682 | C |
| *In(3L)P* | *3L* | 3142231 | A |
| *In(3L)P* | *3L* | 3145702 | T |
| *In(3L)P* | *3L* | 3148304 | A |
| *In(3L)P* | *3L* | 3152282 | C |
| *In(3L)P* | *3L* | 3156337 | A |
| *In(3L)P* | *3L* | 3165913 | A |
| *In(3L)P* | *3L* | 3172232 | A |
| *In(3L)P* | *3L* | 3172572 | A |
| *In(3L)P* | *3L* | 3190585 | G |
| *In(3L)P* | *3L* | 3191474 | G |
| *In(3L)P* | *3L* | 3192621 | A |
| *In(3L)P* | *3L* | 3194000 | T |
| *In(3L)P* | *3L* | 3195095 | A |
| *In(3L)P* | *3L* | 3198656 | T |
| *In(3L)P* | *3L* | 3202276 | G |
| *In(3L)P* | *3L* | 3203140 | T |
| *In(3L)P* | *3L* | 3203449 | G |
| *In(3L)P* | *3L* | 3205464 | C |
| *In(3L)P* | *3L* | 3244232 | A |
| *In(3L)P* | *3L* | 3250267 | C |
| *In(3L)P* | *3L* | 3251643 | G |
| *In(3L)P* | *3L* | 3258888 | A |
| *In(3L)P* | *3L* | 3260348 | A |
| *In(3L)P* | *3L* | 3274254 | C |
| *In(3L)P* | *3L* | 3284533 | A |
| *In(3L)P* | *3L* | 3388479 | G |
| *In(3L)P* | *3L* | 3389696 | A |
| *In(3L)P* | *3L* | 3390222 | G |
| *In(3L)P* | *3L* | 3397051 | A |
| *In(3L)P* | *3L* | 3430131 | G |
| *In(3L)P* | *3L* | 3764444 | T |
| *In(3L)P* | *3L* | 5399565 | T |
| *In(3L)P* | *3L* | 15633845 | G |
| *In(3L)P* | *3L* | 15970961 | G |
| *In(3L)P* | *3L* | 16165187 | A |
| *In(3L)P* | *3L* | 16165189 | T |
| *In(3L)P* | *3L* | 16165230 | C |
| *In(3L)P* | *3L* | 16170296 | C |
| *In(3L)P* | *3L* | 16193541 | A |
| *In(3L)P* | *3L* | 16201506 | G |
| *In(3L)P* | *3L* | 16217175 | A |
| *In(3L)P* | *3L* | 16222536 | G |
| *In(3L)P* | *3L* | 16223154 | C |
| *In(3L)P* | *3L* | 16261646 | T |
| *In(3L)P* | *3L* | 16261672 | A |
| *In(3L)P* | *3L* | 16261695 | T |
| *In(3L)P* | *3L* | 16261726 | T |
| *In(3L)P* | *3L* | 16263247 | C |
| *In(3L)P* | *3L* | 16263588 | T |
| *In(3L)P* | *3L* | 16268717 | T |
| *In(3L)P* | *3L* | 16273480 | G |
| *In(3L)P* | *3L* | 16280796 | G |
| *In(3L)P* | *3L* | 16280798 | A |
| *In(3L)P* | *3L* | 16289482 | C |
| *In(3L)P* | *3L* | 16290594 | G |
| *In(3L)P* | *3L* | 16290972 | T |
| *In(3L)P* | *3L* | 16291332 | G |
| *In(3L)P* | *3L* | 16297916 | A |
| *In(3L)P* | *3L* | 16298085 | A |
| *In(3L)P* | *3L* | 16301520 | A |
| *In(3L)P* | *3L* | 16308563 | C |
| *In(3L)P* | *3L* | 16311425 | C |
| *In(3L)P* | *3L* | 16326362 | T |
| *In(3L)P* | *3L* | 16333526 | A |
| *In(3L)P* | *3L* | 16377449 | A |
| *In(3L)P* | *3L* | 16378572 | T |
| *In(3L)P* | *3L* | 16393822 | C |
| *In(3L)P* | *3L* | 16400709 | G |
| *In(3R)C* | *3R* | 13114726 | T |
| *In(3R)C* | *3R* | 16099151 | G |
| *In(3R)C* | *3R* | 16104479 | A |
| *In(3R)C* | *3R* | 16110028 | T |
| *In(3R)C* | *3R* | 16114832 | G |
| *In(3R)C* | *3R* | 16145902 | G |
| *In(3R)C* | *3R* | 16145903 | T |
| *In(3R)C* | *3R* | 16191928 | T |
| *In(3R)C* | *3R* | 16864615 | C |
| *In(3R)C* | *3R* | 16893226 | C |
| *In(3R)C* | *3R* | 16918188 | T |
| *In(3R)C* | *3R* | 19748559 | A |
| *In(3R)C* | *3R* | 19755935 | T |
| *In(3R)C* | *3R* | 20442534 | G |
| *In(3R)C* | *3R* | 20498606 | G |
| *In(3R)C* | *3R* | 20558459 | G |
| *In(3R)C* | *3R* | 20924283 | T |
| *In(3R)C* | *3R* | 20943910 | T |
| *In(3R)C* | *3R* | 23033890 | A |
| *In(3R)C* | *3R* | 24007045 | T |
| *In(3R)C* | *3R* | 24007371 | G |
| *In(3R)C* | *3R* | 24009461 | T |
| *In(3R)C* | *3R* | 24014066 | T |
| *In(3R)C* | *3R* | 24029634 | T |
| *In(3R)C* | *3R* | 24041884 | A |
| *In(3R)C* | *3R* | 24041990 | T |
| *In(3R)C* | *3R* | 24043681 | C |
| *In(3R)C* | *3R* | 24044393 | A |
| *In(3R)C* | *3R* | 24078020 | G |
| *In(3R)C* | *3R* | 24085873 | T |
| *In(3R)C* | *3R* | 24096291 | T |
| *In(3R)C* | *3R* | 24138943 | G |
| *In(3R)C* | *3R* | 24142235 | C |
| *In(3R)C* | *3R* | 24150589 | T |
| *In(3R)C* | *3R* | 24163991 | T |
| *In(3R)C* | *3R* | 24171563 | A |
| *In(3R)C* | *3R* | 24172382 | A |
| *In(3R)C* | *3R* | 24195591 | G |
| *In(3R)C* | *3R* | 24201208 | T |
| *In(3R)C* | *3R* | 24242753 | A |
| *In(3R)C* | *3R* | 24243280 | C |
| *In(3R)C* | *3R* | 24279617 | G |
| *In(3R)C* | *3R* | 24282605 | T |
| *In(3R)C* | *3R* | 24298461 | A |
| *In(3R)C* | *3R* | 24342811 | T |
| *In(3R)C* | *3R* | 24374212 | G |
| *In(3R)C* | *3R* | 24409151 | A |
| *In(3R)C* | *3R* | 24422474 | C |
| *In(3R)C* | *3R* | 24467871 | T |
| *In(3R)C* | *3R* | 24487712 | G |
| *In(3R)C* | *3R* | 24493367 | G |
| *In(3R)C* | *3R* | 24506558 | G |
| *In(3R)C* | *3R* | 24512937 | T |
| *In(3R)C* | *3R* | 24522397 | G |
| *In(3R)C* | *3R* | 24551095 | A |
| *In(3R)C* | *3R* | 24690673 | T |
| *In(3R)C* | *3R* | 24693933 | A |
| *In(3R)C* | *3R* | 24694365 | A |
| *In(3R)C* | *3R* | 24719313 | A |
| *In(3R)C* | *3R* | 25096252 | A |
| *In(3R)C* | *3R* | 25106453 | C |
| *In(3R)C* | *3R* | 25136719 | A |
| *In(3R)C* | *3R* | 25175337 | A |
| *In(3R)C* | *3R* | 25176234 | G |
| *In(3R)C* | *3R* | 25179516 | G |
| *In(3R)C* | *3R* | 25193278 | A |
| *In(3R)C* | *3R* | 25216865 | A |
| *In(3R)C* | *3R* | 25222529 | G |
| *In(3R)C* | *3R* | 25242597 | G |
| *In(3R)C* | *3R* | 25248195 | T |
| *In(3R)C* | *3R* | 25269879 | A |
| *In(3R)C* | *3R* | 25315158 | A |
| *In(3R)C* | *3R* | 25329587 | C |
| *In(3R)C* | *3R* | 25474612 | T |
| *In(3R)C* | *3R* | 25489586 | C |
| *In(3R)C* | *3R* | 25505585 | C |
| *In(3R)C* | *3R* | 25538313 | A |
| *In(3R)C* | *3R* | 25560925 | A |
| *In(3R)C* | *3R* | 25567683 | C |
| *In(3R)C* | *3R* | 25583469 | A |
| *In(3R)C* | *3R* | 25596484 | T |
| *In(3R)C* | *3R* | 25598648 | C |
| *In(3R)C* | *3R* | 25599170 | T |
| *In(3R)C* | *3R* | 25604540 | T |
| *In(3R)C* | *3R* | 25604725 | C |
| *In(3R)C* | *3R* | 25605392 | G |
| *In(3R)C* | *3R* | 25605428 | T |
| *In(3R)C* | *3R* | 25632833 | A |
| *In(3R)C* | *3R* | 25647947 | C |
| *In(3R)C* | *3R* | 25680387 | G |
| *In(3R)C* | *3R* | 25686401 | C |
| *In(3R)C* | *3R* | 25686744 | A |
| *In(3R)C* | *3R* | 25689415 | G |
| *In(3R)C* | *3R* | 25689478 | T |
| *In(3R)C* | *3R* | 25692175 | T |
| *In(3R)C* | *3R* | 25776627 | C |
| *In(3R)C* | *3R* | 25789208 | A |
| *In(3R)C* | *3R* | 25789641 | C |
| *In(3R)C* | *3R* | 25798811 | A |
| *In(3R)C* | *3R* | 25810959 | T |
| *In(3R)C* | *3R* | 25822138 | T |
| *In(3R)C* | *3R* | 25830799 | T |
| *In(3R)C* | *3R* | 25836339 | T |
| *In(3R)C* | *3R* | 25865969 | A |
| *In(3R)C* | *3R* | 25881149 | A |
| *In(3R)C* | *3R* | 25884722 | G |
| *In(3R)C* | *3R* | 25885398 | A |
| *In(3R)C* | *3R* | 25885568 | A |
| *In(3R)C* | *3R* | 25892882 | T |
| *In(3R)C* | *3R* | 25893312 | T |
| *In(3R)C* | *3R* | 25901563 | T |
| *In(3R)C* | *3R* | 25904049 | C |
| *In(3R)C* | *3R* | 25904085 | G |
| *In(3R)C* | *3R* | 26052763 | T |
| *In(3R)C* | *3R* | 26450277 | C |
| *In(3R)C* | *3R* | 26502830 | A |
| *In(3R)C* | *3R* | 26541828 | C |
| *In(3R)C* | *3R* | 26553123 | T |
| *In(3R)C* | *3R* | 26833261 | T |
| *In(3R)C* | *3R* | 27033799 | A |
| *In(3R)C* | *3R* | 27050399 | C |
| *In(3R)C* | *3R* | 27050401 | G |
| *In(3R)C* | *3R* | 27183127 | A |
| *In(3R)C* | *3R* | 27187114 | G |
| *In(3R)C* | *3R* | 27189512 | G |
| *In(3R)C* | *3R* | 27213181 | T |
| *In(3R)C* | *3R* | 27230179 | G |
| *In(3R)C* | *3R* | 27255032 | G |
| *In(3R)C* | *3R* | 27348805 | A |
| *In(3R)C* | *3R* | 27350380 | T |
| *In(3R)C* | *3R* | 27355100 | A |
| *In(3R)C* | *3R* | 27355101 | T |
| *In(3R)C* | *3R* | 27367655 | T |
| *In(3R)C* | *3R* | 27376219 | A |
| *In(3R)C* | *3R* | 27450892 | T |
| *In(3R)C* | *3R* | 27536048 | G |
| *In(3R)C* | *3R* | 27560508 | G |
| *In(3R)C* | *3R* | 27560856 | A |
| *In(3R)C* | *3R* | 27561118 | A |
| *In(3R)C* | *3R* | 27813043 | T |
| *In(3R)C* | *3R* | 27815314 | C |
| *In(3R)C* | *3R* | 27819657 | C |
| *In(3R)C* | *3R* | 27873302 | A |
| *In(3R)C* | *3R* | 27885889 | A |
| *In(3R)K* | *3R* | 7569591 | G |
| *In(3R)K* | *3R* | 7587158 | A |
| *In(3R)K* | *3R* | 7763547 | T |
| *In(3R)K* | *3R* | 21961212 | C |
| *In(3R)Mo* | *3R* | 15955370 | C |
| *In(3R)Mo* | *3R* | 15956205 | G |
| *In(3R)Mo* | *3R* | 16012652 | A |
| *In(3R)Mo* | *3R* | 16054389 | T |
| *In(3R)Mo* | *3R* | 16088352 | T |
| *In(3R)Mo* | *3R* | 16101901 | A |
| *In(3R)Mo* | *3R* | 16309968 | A |
| *In(3R)Mo* | *3R* | 16310458 | T |
| *In(3R)Mo* | *3R* | 16321720 | G |
| *In(3R)Mo* | *3R* | 16324886 | T |
| *In(3R)Mo* | *3R* | 16327977 | A |
| *In(3R)Mo* | *3R* | 16329725 | C |
| *In(3R)Mo* | *3R* | 16354768 | T |
| *In(3R)Mo* | *3R* | 16358463 | A |
| *In(3R)Mo* | *3R* | 16477118 | A |
| *In(3R)Mo* | *3R* | 16505890 | C |
| *In(3R)Mo* | *3R* | 16563347 | C |
| *In(3R)Mo* | *3R* | 16564891 | A |
| *In(3R)Mo* | *3R* | 16565899 | T |
| *In(3R)Mo* | *3R* | 16825891 | A |
| *In(3R)Mo* | *3R* | 16840241 | T |
| *In(3R)Mo* | *3R* | 16877262 | C |
| *In(3R)Mo* | *3R* | 16881477 | A |
| *In(3R)Mo* | *3R* | 16882614 | C |
| *In(3R)Mo* | *3R* | 16914806 | G |
| *In(3R)Mo* | *3R* | 17081985 | T |
| *In(3R)Mo* | *3R* | 17145087 | G |
| *In(3R)Mo* | *3R* | 17161903 | T |
| *In(3R)Mo* | *3R* | 17183342 | T |
| *In(3R)Mo* | *3R* | 17190382 | C |
| *In(3R)Mo* | *3R* | 17203074 | T |
| *In(3R)Mo* | *3R* | 17226102 | G |
| *In(3R)Mo* | *3R* | 17231109 | T |
| *In(3R)Mo* | *3R* | 17252528 | A |
| *In(3R)Mo* | *3R* | 17255885 | A |
| *In(3R)Mo* | *3R* | 17257625 | A |
| *In(3R)Mo* | *3R* | 17261973 | C |
| *In(3R)Mo* | *3R* | 17346744 | A |
| *In(3R)Mo* | *3R* | 17482849 | T |
| *In(3R)Mo* | *3R* | 17492333 | T |
| *In(3R)Mo* | *3R* | 17512751 | T |
| *In(3R)Mo* | *3R* | 17543357 | A |
| *In(3R)Mo* | *3R* | 17570809 | A |
| *In(3R)Mo* | *3R* | 17574820 | T |
| *In(3R)Mo* | *3R* | 17575776 | T |
| *In(3R)Mo* | *3R* | 17614569 | T |
| *In(3R)Mo* | *3R* | 17618094 | T |
| *In(3R)Mo* | *3R* | 17653963 | A |
| *In(3R)Mo* | *3R* | 17673637 | T |
| *In(3R)Mo* | *3R* | 17731781 | T |
| *In(3R)Mo* | *3R* | 17752308 | T |
| *In(3R)Mo* | *3R* | 17775264 | A |
| *In(3R)Mo* | *3R* | 17798722 | T |
| *In(3R)Mo* | *3R* | 17812150 | A |
| *In(3R)Mo* | *3R* | 17812763 | A |
| *In(3R)Mo* | *3R* | 17833454 | T |
| *In(3R)Mo* | *3R* | 17871386 | A |
| *In(3R)Mo* | *3R* | 17878212 | T |
| *In(3R)Mo* | *3R* | 17893124 | G |
| *In(3R)Mo* | *3R* | 17900659 | A |
| *In(3R)Mo* | *3R* | 17905561 | T |
| *In(3R)Mo* | *3R* | 17909484 | G |
| *In(3R)Mo* | *3R* | 17914642 | A |
| *In(3R)Mo* | *3R* | 17915717 | C |
| *In(3R)Mo* | *3R* | 18018705 | C |
| *In(3R)Mo* | *3R* | 18110219 | T |
| *In(3R)Mo* | *3R* | 18151777 | T |
| *In(3R)Mo* | *3R* | 18195302 | T |
| *In(3R)Mo* | *3R* | 18227258 | T |
| *In(3R)Mo* | *3R* | 18229705 | C |
| *In(3R)Mo* | *3R* | 18236474 | C |
| *In(3R)Mo* | *3R* | 18237459 | A |
| *In(3R)Mo* | *3R* | 18248909 | G |
| *In(3R)Mo* | *3R* | 18405781 | T |
| *In(3R)Mo* | *3R* | 18747568 | T |
| *In(3R)Mo* | *3R* | 18755175 | G |
| *In(3R)Mo* | *3R* | 19051282 | T |
| *In(3R)Mo* | *3R* | 19310873 | A |
| *In(3R)Mo* | *3R* | 19540597 | C |
| *In(3R)Mo* | *3R* | 19573177 | T |
| *In(3R)Mo* | *3R* | 19604547 | T |
| *In(3R)Mo* | *3R* | 19614762 | A |
| *In(3R)Mo* | *3R* | 19616872 | T |
| *In(3R)Mo* | *3R* | 19619722 | G |
| *In(3R)Mo* | *3R* | 19621728 | A |
| *In(3R)Mo* | *3R* | 19625953 | T |
| *In(3R)Mo* | *3R* | 19686653 | A |
| *In(3R)Mo* | *3R* | 19690483 | T |
| *In(3R)Mo* | *3R* | 19928635 | C |
| *In(3R)Mo* | *3R* | 20090826 | G |
| *In(3R)Mo* | *3R* | 20102331 | G |
| *In(3R)Mo* | *3R* | 20106419 | T |
| *In(3R)Mo* | *3R* | 20108509 | G |
| *In(3R)Mo* | *3R* | 20712447 | A |
| *In(3R)Mo* | *3R* | 20717876 | G |
| *In(3R)Mo* | *3R* | 20720722 | C |
| *In(3R)Mo* | *3R* | 20761490 | T |
| *In(3R)Mo* | *3R* | 20809103 | T |
| *In(3R)Mo* | *3R* | 20815949 | C |
| *In(3R)Mo* | *3R* | 20837056 | A |
| *In(3R)Mo* | *3R* | 21380190 | A |
| *In(3R)Mo* | *3R* | 21807559 | A |
| *In(3R)Mo* | *3R* | 21956164 | G |
| *In(3R)Mo* | *3R* | 22035252 | T |
| *In(3R)Mo* | *3R* | 22399475 | A |
| *In(3R)Mo* | *3R* | 22436302 | C |
| *In(3R)Mo* | *3R* | 22477725 | G |
| *In(3R)Mo* | *3R* | 22635953 | T |
| *In(3R)Mo* | *3R* | 22660660 | T |
| *In(3R)Mo* | *3R* | 22661217 | A |
| *In(3R)Mo* | *3R* | 22703601 | C |
| *In(3R)Mo* | *3R* | 22850222 | A |
| *In(3R)Mo* | *3R* | 23028130 | G |
| *In(3R)Mo* | *3R* | 23504771 | A |
| *In(3R)Mo* | *3R* | 23589504 | C |
| *In(3R)Mo* | *3R* | 24757430 | T |
| *In(3R)Mo* | *3R* | 24834927 | A |
| *In(3R)Mo* | *3R* | 25052744 | T |
| *In(3R)Mo* | *3R* | 25065632 | T |
| *In(3R)Mo* | *3R* | 25087248 | G |
| *In(3R)Mo* | *3R* | 25206657 | T |
| *In(3R)Mo* | *3R* | 25250616 | A |
| *In(3R)Mo* | *3R* | 25253902 | A |
| *In(3R)Mo* | *3R* | 25293082 | T |
| *In(3R)Mo* | *3R* | 25354278 | T |
| *In(3R)Mo* | *3R* | 25687897 | G |
| *In(3R)Mo* | *3R* | 26584256 | T |
| *In(3R)Mo* | *3R* | 26725477 | A |
| *In(3R)Mo* | *3R* | 26930971 | C |
| *In(3R)Mo* | *3R* | 26933596 | C |
| *In(3R)Mo* | *3R* | 26949382 | A |
| *In(3R)Mo* | *3R* | 26955397 | C |
| *In(3R)Mo* | *3R* | 26960620 | T |
| *In(3R)Mo* | *3R* | 27080067 | G |
| *In(3R)Mo* | *3R* | 27091763 | A |
| *In(3R)Mo* | *3R* | 27114289 | A |
| *In(3R)Mo* | *3R* | 27124527 | T |
| *In(3R)Mo* | *3R* | 27136784 | C |
| *In(3R)Mo* | *3R* | 27266479 | A |
| *In(3R)Mo* | *3R* | 27382123 | C |
| *In(3R)Mo* | *3R* | 27395403 | C |
| *In(3R)Mo* | *3R* | 27395667 | A |
| *In(3R)Mo* | *3R* | 27396540 | T |
| *In(3R)Mo* | *3R* | 27396541 | T |
| *In(3R)Mo* | *3R* | 27419936 | A |
| *In(3R)Mo* | *3R* | 27430813 | G |
| *In(3R)Mo* | *3R* | 27434102 | T |
| *In(3R)Mo* | *3R* | 27434183 | G |
| *In(3R)Mo* | *3R* | 27434363 | C |
| *In(3R)Mo* | *3R* | 27438021 | T |
| *In(3R)Payne* | *3R* | 12257883 | G |
| *In(3R)Payne* | *3R* | 12259133 | C |
| *In(3R)Payne* | *3R* | 12259894 | A |
| *In(3R)Payne* | *3R* | 12263816 | C |
| *In(3R)Payne* | *3R* | 12289495 | C |
| *In(3R)Payne* | *3R* | 12298324 | A |
| *In(3R)Payne* | *3R* | 12298456 | T |
| *In(3R)Payne* | *3R* | 12316508 | C |
| *In(3R)Payne* | *3R* | 17442150 | T |
| *In(3R)Payne* | *3R* | 20343494 | A |
| *In(3R)Payne* | *3R* | 20562004 | T |
| *In(3R)Payne* | *3R* | 20567442 | G |
| *In(3R)Payne* | *3R* | 20567659 | C |
| *In(3R)Payne* | *3R* | 20567832 | C |
| *In(3R)Payne* | *3R* | 20575824 | G |
| *In(3R)Payne* | *3R* | 20580991 | A |
| *In(3R)Payne* | *3R* | 20580995 | T |
| *In(3R)Payne* | *3R* | 20590675 | G |
| *In(3R)Payne* | *3R* | 20591144 | A |

Supporting Table 5. Inversion frequencies during the experimental evolution experiment. Inversion frequencies estimated from Pool-Seq data using inversion-specific SNP markers in our laboratory natural selection experiment. Shown are median and average (in parentheses) of allele frequencies for each population.

| **Generation** | **Treatment** | **Replicate** | ***In(2L)t*** | ***In(2R)Ns*** | ***In(3L)P*** | ***In(3R)C*** | ***In(3R)K*** | ***In(3R)Mo*** | ***In(3R)P*** |
| --- | --- | --- | --- | --- | --- | --- | --- | --- | --- |
| 0 |  | 1 | 0.39 (0.43) | 0.1 (0.11) | 0.16 (0.12) | 0.16 (0.17) | 0.01 (0.01) | 0.04 (0) | 0.21 (0.21) |
| 0 |  | 2 | 0.39 (0.31) | 0.09 (0.1) | 0.15 (0.25) | 0.15 (0.16) | 0.03 (0.06) | 0.05 (0.08) | 0.19 (0.18) |
| 0 |  | 3 | 0.43 (0.45) | 0.09 (0.08) | 0.14 (0.13) | 0.15 (0.09) | 0.01 (0) | 0.05 (0.04) | 0.19 (0.17) |
| 15 | hot | 1 | 0.51 (0.56) | 0.05 (0.06) | 0.12 (0.07) | 0.38 (0.57) | 0.02 (0) | 0.08 (0.07) | 0.06 (0.18) |
| 37 | hot | 1 | 0.39 (0.44) | 0.02 (0) | 0.13 (0.2) | 0.41 (0.34) | 0 (0) | 0.06 (0.06) | 0.02 (0.02) |
| 59 | hot | 1 | 0.25 (0.34) | 0 (0) | 0.05 (0.04) | 0.48 (0.5) | 0 (0) | 0.05 (0.05) | 0 (0.04) |
| 15 | hot | 2 | 0.43 (0.44) | 0.03 (0.03) | 0.25 (0.19) | 0.36 (0.25) | 0 (0) | 0.04 (0) | 0.07 (0.02) |
| 37 | hot | 2 | 0.25 (0.12) | 0 (0) | 0.12 (0.31) | 0.36 (0.27) | 0 (0) | 0.03 (0) | 0.02 (0) |
| 59 | hot | 2 | 0.25 (0.28) | 0 (0) | 0.03 (0) | 0.27 (0.24) | 0 (0) | 0.07 (0.05) | 0.01 (0.01) |
| 15 | hot | 3 | 0.52 (0.5) | 0.06 (0.04) | 0.22 (0.22) | 0.29 (0.34) | 0 (0) | 0.17 (0.11) | 0.02 (0.01) |
| 27 | hot | 3 | 0.32 (0.22) | 0.04 (0.03) | 0.16 (0.09) | 0.37 (0.16) | 0 (0) | 0.12 (0.11) | 0.02 (0.06) |
| 37 | hot | 3 | 0.37 (0.37) | 0.03 (0.02) | 0.01 (0.05) | 0.39 (0.3) | 0 (0) | 0.1 (0.1) | 0.01 (0.09) |
| 59 | hot | 3 | 0.23 (0.21) | 0 (0) | 0 (0) | 0.5 (0.61) | 0 (0) | 0.01 (0) | 0 (0.02) |
| 15 | cold | 1 | 0.21 (0.21) | 0.01 (0.01) | 0.11 (0.08) | 0.05 (0.08) | 0 (0) | 0.21 (0.16) | 0.19 (0.22) |
| 33 | cold | 1 | 0.39 (0.39) | 0 (0) | 0.07 (0.07) | 0.07 (0.07) | 0 (0) | 0.22 (0.13) | 0.03 (0.03) |
| 15 | cold | 2 | 0.42 (0.42) | 0.06 (0.02) | 0.12 (0.2) | 0.08 (0.06) | 0 (0) | 0.2 (0.18) | 0.07 (0.07) |
| 33 | cold | 2 | 0.21 (0.14) | 0.01 (0.03) | 0.11 (0.12) | 0.16 (0.09) | 0 (0) | 0.24 (0.24) | 0 (0) |
| 15 | cold | 3 | 0.39 (0.39) | 0.09 (0.09) | 0.05 (0.11) | 0.11 (0.03) | 0 (0) | 0.23 (0.28) | 0.06 (0.04) |
| 33 | cold | 3 | 0.56 (0.52) | 0.02 (0) | 0.07 (0.04) | 0.15 (0.15) | 0 (0) | 0.28 (0.35) | 0 (0) |

**Supporting Table 6. Inversion frequency differences during experimental evolution.** *P*-values from CMH tests performed between the base population and consecutive generations during the experimental evolution experiment. *P*-values were combined by averaging across all marker SNPs for each inversion.

| **Inversion** | **0_15_hot** | **0_37_hot** | **0_59_hot** | **0_15_cold** | **0_33_cold** |
| --- | --- | --- | --- | --- | --- |
| *In(2L)t* | 0.3259 | 0.4464 | 0.0739 | 0.3081 | 0.5377 |
| *In(2R)NS* | 0.3757 | 0.1298 | 0.0139 | 0.3150 | 0.0209 |
| *In(3L)P* | 0.4246 | 0.2829 | 0.0032 | 0.3877 | 0.2022 |
| *In(3R)C* | 0.0275 | 0.0129 | 0.0012 | 0.2040 | 0.3445 |
| *In(3R)K* | 0.4080 | 0.4394 | 0.2045 | 0.4543 | 0.1755 |
| *In(3R)Mo* | 0.2035 | 0.4997 | 0.4699 | 0.0232 | 0.0071 |
| *In(3R)Payne* | 0.0048 | 0.0132 | 0.0009 | 0.0639 | 0.0000 |

Supporting Table 7. Inversion frequencies in natural populations. Inversion frequencies estimated from Pool-Seq data using inversion-specific SNP markers for the Australian (Kolaczkowski *et al*. 2011) and North American (Fabian *et al*. 2012) data. Median and average (in parentheses) of allele frequencies for each population.

|  | ***In(2L)t*** | ***In(2R)Ns*** | ***In(3L)P*** | ***In(3R)C*** | ***In(3R)K*** | ***In(3R)Mo*** | ***In(3R)Payne*** |
| --- | --- | --- | --- | --- | --- | --- | --- |
| **Florida** | 0.41 (0.38) | 0.01 (0.01) | 0.09 (0.09) | 0.01 (0) | 0 (0) | 0 (0) | 0.49 (0.54) |
| **Pennsylvania** | 0.23 (0.22) | 0.04 (0.05) | 0.01 (0.05) | 0.01 (0) | 0 (0.01) | 0.08 (0.05) | 0.02 (0.06) |
| **Maine** | 0.2 (0.21) | 0.1 (0.11) | 0 (0.04) | 0.02 (0.02) | 0.06 (0.07) | 0.14 (0.14) | 0.01 (0) |
| **Queensland** | 0.2 (0.38) | 0.05 (0.04) | 0.09 (0.08) | 0 (0) | 0 (0) | 0 (0) | 0.23 (0.13) |
| **Tasmania** | 0 (0) | 0 (0) | 0 (0) | 0 (0) | 0 (0) | 0 (0) | 0.05 (0) |

**Supporting Table 8. Inversion frequency differences in natural populations.**

*P*-values from Fisher Exact Tests (FET) performed between the lowest-latitude population (Florida and Queensland, respectively) and all other populations in North America (Florida-Pennsylvania: FP; Florida-Maine: FM) and Australia (Queensland-Tasmania: QT) (also see Kolaczkowski *et al*. 2011; Fabian *et al.* 2012). *P*-values were combined by averaging across all marker SNPs for each inversion.

| **Inversion** | **FP** | **FM** | **QT** |
| --- | --- | --- | --- |
| *In(2L)t* | 0.1848 | 0.0220 | 0.4987 |
| *In(2R)Ns* | 0.2692 | 0.0703 | 0.6332 |
| *In(3L)P* | 0.1172 | 0.0752 | 0.5460 |
| *In(3R)C* | 0.2043 | 0.3590 | 0.6584 |
| *In(3R)K* | 0.2500 | 0.1091 | 1.0000 |
| *In(3R)Mo* | 0.0853 | 0.0089 | 0.7476 |
| *In(3R)Payne* | 0.0000 | 0.0000 | 0.3516 |

**Supporting Table 9. Expected inversion frequency changes due to neutral evolution.** Here, we performed 100,000 simulations of inversion frequency changes as expected due to genetic drift based on a Wright-Fisher model and tested whether the changes were in the expected direction (sign of frequency change) and stronger than observed in the real data. The empirical *P*-value corresponds to the proportion of simulations resulting in stronger inversion frequency changes consistent across all replicates than observed in the real data from the laboratory natural selection experiment. Note that the frequency increases of *In(3R)C* in the hot and *In(3R)Mo* in the cold temperature treatment were significantly higher than expected due to genetic drift (*P*-value < 0.0042; Bonferroni corrected α of 0.05). Additionally, the frequency of *In(3R)P* significantly decreased stronger than expected due to neutral evolution in the cold temperature treatment. All significant results are indicated by an asterisk.

| **Inversion** | **Treatment** | **Generations simulated** | **Sign of frequency change** | **Empirical *P*-value** |
| --- | --- | --- | --- | --- |
| *In(2L)t* | cold | 33 | - | 0.2105 |
| *In(2L)t* | hot | 59 | - | 0.0302 |
| *In(2R)NS* | cold | 33 | - | 0.0577 |
| *In(2R)NS* | hot | 59 | - | 0.1352 |
| *In(3L)P* | cold | 33 | - | 0.0994 |
| *In(3L)P* | hot | 59 | - | 0.0821 |
| *In(3R)C* | cold | 33 | - | 0.2033 |
| *In(3R)C* | hot | 59 | + | 0.0031^*^ |
| *In(3R)Mo* | cold | 33 | + | 0.0002^*^ |
| *In(3R)Mo* | hot | 59 | - | 0.5250 |
| *In(3R)P* | cold | 33 | - | 0.0020^*^ |
| *In(3R)P* | hot | 59 | - | 0.0152 |

**Supporting Table 10. Reliability of inversion frequency estimates.** *P*-values of FET tests used to test for significant differences between empirically determined inversion frequencies (via karyotyping) and those estimated from inversion-specific SNP markers. *P*-values were. Note that non of the *P*-values were significant, indicating that the two methods for estimating inversion frequencies did not differ from each other in their reliability.

| **Generation** | **Regime** | **Rep** | ***In(2L)t*** | ***In(2R)Ns*** | ***In(3L)P*** | ***In(3R)C*** | ***In(3R)K*** | ***In(3R)Mo*** | ***In(3R)P*** |
| --- | --- | --- | --- | --- | --- | --- | --- | --- | --- |
| 59 | hot | 1 | 0.29 | 1.00 | 1.00 | 1.00 | 1.00 | 1.00 | 1.00 |
| 59 | hot | 2 | 0.82 | 1.00 | 0.34 | 0.42 | 1.00 | 0.66 | 0.12 |
| 59 | hot | 3 | 0.08 | 1.00 | 1.00 | 0.44 | 1.00 | 1.00 | 1.00 |
| 33 | cold | 1 | 1.00 | 1.00 | 0.72 | 1.00 | 1.00 | 1.00 | 0.14 |
| 33 | cold | 2 | 0.31 | 1.00 | 0.33 | 0.16 | 1.00 | 0.83 | 1.00 |
| 33 | cold | 3 | 0.26 | 0.17 | 0.34 | 0.76 | 1.00 | 0.48 | 1.00 |

Supporting Table 11. Allele sharing among karyotypes. Amount of allele sharing between individuals (numbers 96 and 100) carrying *In(3R)Mo* and individuals with other chromosomal arrangements. We only used SNPs which were polymorphic between individuals 96 and 100 and the other *In(3R)Mo* chromosomes, located in two polymorphic regions within the inversion boundaries; region 1 spanned positions 17,300,000 to 19,400,000 and region 2 positions 23,400,000 to 24,200,000.

| **Chrom. region** | **Individual** | **No. of SNPs** | ***In(3R)C*** | ***In(3R)Payne*** | **Standard** |
| --- | --- | --- | --- | --- | --- |
| 1 | 96 | 382 | 63.97% | 47.00% | 100.00% |
| 1 | 100 | 1197 | 73.77% | 48.12% | 78.11% |
| 2 | 96 | 374 | 64.97% | 56.15% | 100.00% |

Supporting Table 12. Statistical power of inversion-specific marker alleles in estimating inversion frequencies. Exact *P*-values obtained by sampling from a *χ*^2^-distribution calculated from randomly drawn SNPs by means of CMH tests. Significant *P*-values (*P* < 0.05) indicate that inversion-specific markers performed better than SNPs randomly drawn from within the inversion body.

| **Inversion** | **0_15_hot** | **0_37_hot** | **0_59_hot** | **0_15_cold** | **0_33_cold** |
| --- | --- | --- | --- | --- | --- |
| *In(2L)t* | 0.2628 | 0.9400 | 0.0730 | 0.6709 | 0.9997 |
| *In(2R)NS* | 0.6501 | 0.0812 | 0.0000 | 0.8527 | 0.0000 |
| *In(3L)P* | 0.9989 | 0.9881 | 0.0003 | 0.5320 | 0.2976 |
| *In(3R)C* | 0.0000 | 0.0000 | 0.0000 | 0.0802 | 1.0000 |
| *In(3R)K* | 0.9727 | 0.9775 | 0.9711 | 0.9039 | 0.8684 |
| *In(3R)Mo* | 0.6842 | 1.0000 | 1.0000 | 0.0000 | 0.0000 |
| *In(3R)Payne* | 0.0000 | 0.0001 | 0.0000 | 0.0089 | 0.0000 |

**Documentation of bioinformatics pipeline**

See Supporting Folder 1 (downloadable zip file) for Python scripts and their description.
